# Microbial consortium mediated acceleration of the defense response in potato against *Alternaria solani* through prodigious inflation in phenylpropanoid derivatives and redox homeostasis

**DOI:** 10.1016/j.heliyon.2023.e22148

**Published:** 2023-11-10

**Authors:** Sumit Kumar, Ram Chandra, Lopamudra Behera, Ichini Sudhir, Mukesh Meena, Shailendra Singh, Chetan Keswani

**Affiliations:** a Department of Mycology and Plant Pathology, Institute of Agricultural Sciences, Banaras Hindu University, Varanasi, 221005, India; b Department of Plant Pathology, B.M. College of Agriculture, Khandwa, Rajmata Vijayaraje Scindia Krishi Vishwa Vidyalaya, Gwalior, 474002, India; c Laboratory of Phytopathology and Microbial Biotechnology, Department of Botany, University Collage of Science, Mohanlal Sukhadia University, Udaipur, 313001, India; d Department of Biotechnology, Invertis University, Bareilly, 243123, India; e Academy of Biology and Biotechnology, Southern Federal University, Rostov-on-Don, 344090, Russia

**Keywords:** Microbial consortium, Induced systemic resistance, *Alternaria solani*, Antioxidants, Polyphenolics, Biopriming, Seed treatment, Plant stress, Plant health, Sustainable agriculture

## Abstract

The present study was carried out in a pot experiment to examine the bioefficacy of three biocontrol agents, viz., *Trichoderma viride*, *Bacillus subtilis*, and *Pseudomonas fluorescens*, either alone or in consortium, on plant growth promotion and activation of defense responses in potato against the early blight pathogen *Alternaria solani*. The results demonstrate significant enhancement in growth parameters in plants bioprimed with the triple-microbe consortium compared to other treatments. In potato, the disease incidence percentage was significantly reduced in plants treated with the triple-microbe consortium compared to untreated control plants challenged with *A. solani*. Potato tubers treated with the consortium and challenged with pathogen showed significant activation of defense-related enzymes such as peroxidase (PO) at 96 h after pathogen inoculation (hapi) while, both polyphenol oxidase (PPO), and phenylalanine ammonia-lyase (PAL) at 72 hapi, compared to the individual and dual microbial consortia-treated plants. The expression of antioxidant enzymes like superoxide dismutase (SOD) and catalase (CAT) and the accumulation of pathogenesis-related proteins such as chitinase and β-1,3-glucanase were observed to be highest at 72 hapi in the triple microbe consortium as compared to other treatments. HPLC analysis revealed significant induction in polyphenolic compounds in triple-consortium bioprimed plants compared to the control at 72 hapi. Histochemical analysis of hydrogen peroxide (H_2_O_2_) clearly showed maximum accumulation of H_2_O_2_ in pathogen-inoculated control plants, while the lowest was observed in triple-microbe consortium at 72 hapi. The findings of this study suggest that biopriming with a microbial consortium improved plant growth and triggered defense responses against *A. solani* through the induction of systemic resistance via modulation of the phenylpropanoid pathway and antioxidative network.

## 1 Introduction

The tuberous crop potato (*Solanum tuberosum* L.) is a key member of the Solanaceae family and one of the most lucrative cash crops in the world [1,2]. Because it is a copious source of natural immunity-enhancing biomolecules, it is considered the “King of vegetables”. Potato cultivation has been difficult in recent years due to biotic and abiotic issues that limit viable yields. Early blight, incited by *Alternaria solani*, is the most important biotic factor and the most devastating fungal infection in all potato and tomato-growing nations, especially India [3,4]. The infamous necrotrophic pathogen *A. solani* causes formidable diseases in vitally important crops belonging to the Solanaceae family, growing under greenhouse and field conditions [5,6]. The large-spored pathogen *A. solani* produces typical symptoms characterized by concentric rings on leaves [7,8]. The devastating pathogen *A. solani* might cause severe yield losses of up to 80 % as reported in India [9,10]. However, integrated disease management (IDM) practices comprised of physical, chemical, biological, cultural, and mechanical control methods have been used to control early blight. The increased dilemma has been achieved with the use of synthetic fungicides on a regular basis; nevertheless, the massive amount of chemicals used produces serious difficulties for the ecosystem and human health, as well as pathogen resistance [[11], [12], [13]]. Although the development of resistant varieties may serve as an eco-friendly approach to manage this disease but the impact is not long-lasting, apart from being time-consuming and expensive [14]. Revealing an ecologically sound and environmentally safe approach for enhancing defense response in potato plants can be adopted to develop a novel management strategy. Microbes-mediated alleviation strategies resolve the problem of resistance development in most cultivated crops and are suitable management practices against detrimental plant microbes [15,16].

Protection of cultivated crops by the elevation of systemic resistance is a promising and holistic method for suppressing a wide range of hostile plant microorganisms in the current context [17]. The application of a suitable microbeial consortium, which consists of a diverse range of microorganisms, can provoke agricultural plant resistance in response to invading diseases. To enhance the efficiency of individual biocontrol agents, combinations of multiple BCAs as well as a group of different microbes can be used, which may perform better than single microorganism by reducing the growth and development of plant diseases in greenhouse and field conditions [18,19]. A combination of different compatible BCAs can induce synergistic action, which may perform better under biotic stress. In the last few decades, more research on microbial consortium has been conducted by scientific communities. With the help of prospective consortia, increased plant growth, development, and induction of defence enzymes and antioxidant enzyme activities, as well as better disease protection, have been reported in vitally important crops such as potato, tomato, pepper, wheat, rice, pea, and chickpea [[20], [21], [22]].

Plant growth-promoting rhizobacterium (PGPR), for instance *Pseudomonas fluorescens*, *Bacillus subtilis* and plant growth-promoting fungi (PGPF), for instance *Trichoderma viride* is used as a potential and fetching biocontrol agent in combating a wide range of devastating plant pathogens [[23], [24], [25], [26], [27]]. The well-documented potential antagonistic ability of these beneficial bacteria and fungi not only works against plant pathogens but also aids in plant growth through various mechanisms such as siderophore formation, root colonization, accumulation of secondary metabolites, uptake of beneficial plant nutrients, and enhanced plant defence against abiotic and biotic factors [[28], [29], [30]]. Potential BCAs work through signaling molecules to increase the production of cell wall degrading enzymes, the biosynthesis of valuable microbes, growth reducing secondary metabolites, antibiosis, and lignin deposition in the cell wall, suppress disease severity, and elevate induced resistance in crop plants [31,32]. *Trichoderma* spp. are soil-borne and belongs to the genus of filamentous fungi, which can employ both direct and indirect biocontrol mechanisms, including biosynthesis of antimicrobial compounds, competition for nutrients and space, and induction of systemic resistance [[33], [34], [35], [36]]. *Trichoderma* spp. can also activate induced systemic resistance (ISR) along with the elevation of defense genes involved in signaling pathways of jasmonic acid or ethylene, which play an important role in conferring resistance to plant pathogens [[37], [38], [39]]. *B*. *subtilis* is an omnipresent Gram-negative bacterium and has the potential to reduce the growth and development of root pathogens via endophytic colonization and the production of broad-spectrum antibiotics [[40], [41], [42]].

Plants are well-equipped against pathogen infection, with a diverse range of defence systems. The rapid generation of reactive oxygen species (ROS) is the host plant's first line of defense [43,44]. Early plant defence responses against pathogen invasion include the formation of ROS such as O_2_^−^, HO^−^, and H_2_O_2_ [45]. To protect themselves from the harmful effects of ROS, plants have a possible antioxidant protection system for detoxification of the detrimental impact of ROS and maintaining the lowest impacts of ROS inside the cell to protect themselves from the toxic effects of ROS [46]. Activation of protective systems consisted of enzymatic and non-enzymatic antioxidants. Among enzymatic antioxidants are superoxide dismutase (SOD), ascorbate peroxidase (APX), catalase (CAT), glutathione reductase (GR), glutathione-S-transferase (GST), guaiacol peroxidase (GPX), and various types of plant peroxidases (POX). Crucial non-enzymatic plant antioxidants, including ascorbate, proline, glutathione, carotenoids, flavonoids, tocopherols, and some other plant phenolic compounds, are also involved in ROS metabolism in response to pathogen attack [47]. Plants treated with beneficial PGPB and PGPF mediate ISR in response to pathogen infection [48]. The ISR defense system has gained substantial attention as a valuable response in plants when plants show elevated defensive capacity with respect to external elicitors. Jasmonic acid and ethylene are the key components of ISR expressed when plants treated with PGPM induce defense-related enzymes, for instance, phenylalanine ammonia-lyase (PAL) and pathogenesis-related (PR) proteins [49], and ROS-scavenging enzymes such as SOD and CAT, along with the synthesis of phenols and flavonoids, which prevent infection incited by phytopathogens [[50], [51], [52]].

Elicitors are a group of bioactive compounds or molecules that act on plants and enhance their resistance. These molecules help in the activation of systemic acquired resistance or induced systemic resistance in host plants through the induction of expression of the pathogenesis-related (PR) genes [53,54]. Among elicitors, fungal elicitors are derived from fungal cell extracts or secretions. Fungal elicitors can rapidly induce the expression of specific genes in plants, which ultimately leads to the activation of secondary metabolic pathways [55]. Fungal elicitors are biologically active substances or chemical signal molecules such as chitin, polysaccharides, glycoproteins, fatty acids, peptides, etc [56,57]. Seed treatment with fungal elicitors enhances the resistance of plants against pathogen infection through the synthesis and accumulation of defense-related enzymes [58,59]. It was reported by De Britto and Jogaiah [60] that broccoli seeds treated with *Trichoderma*-derived trehalose elicitor significantly enhanced systemic resistance against broccoli leaf spot disease by enhancing the production of chitinase and catalase enzymes. In the context of the priming effect, this is an aspect of the resistance induction phenomenon in which, after an elicitor treatment, the mRNAs of plant defense mechanisms are produced, but they will translate to proteins only after pathogen infection. The induced plant uses a priming effect to reduce the fitness costs of resistance induction.

Currently, there is only a limited amount of information known about the notable usage of a suitable microbial consortium for the suppression of early blight disease in potatoes and plant growth promotion activities. The major objectives of the current study were to evaluate the beneficial impacts of suitable *T*. *viride*, *B*. *subtilis*, and *P*. *fluorescens* consortia application on potato plants, taking into account the beneficial qualities of microbial consortium application. The effect of consortium on plant growth and ISR against *A. solani* in the control of early blight of potato was also investigated.

## 2 Materials and methods

### 2.1 Microbial strains

The culture of the fungal biocontrol agent *T. viride* was also obtained from the Indian Type Culture Collection, New Delhi, India (ITCC Accession No. 7057). The antagonistic culture was revived on Trichoderma Selective Medium (TSM) and stored at 4 °C until further use. The well-identified and characterized cultures of bacterial bioagents, viz., *P. fluorescens* (OKC; GenBank Accession JN128891) and *B. subtilis* (BHHU100; GenBank Accession No. JN099686), were obtained from the culture repository of the Plant Health Clinic, Department of Mycology and Plant Pathology, Institute of Agricultural Sciences, Banaras Hindu University, Varanasi, Uttar Pradesh, India. The pure cultures of *P. fluorescens* and *B. subtilis* were maintained on King's B (KB) and nutrient agar (NA) medium slants, respectively.

### 2.2 Inoculum preparation of biocontrol agents

For *B. subtilis* and *P. fluorescens*, a single colony was transferred to 500 ml sterile flasks containing 250 ml of nutrition broth (NB), which were cultured at 28 ± 2 °C for 48 h before being centrifuged at 6000 rpm for 15 min at 4 °C and washed with distilled water. Using a haemocytometer, the inoculum was resuspended in a small quantity of sterile distilled water, and the final concentration was adjusted to 4 × 10^8^ CFU/ml [61]. *Trichoderma viride* was cultivated on potato dextrose agar plates for 6 days at 27 ± 2 °C under fluorescent illumination with a 12 h alternating light and dark cycle. Spores were suspended in sterile distilled water and adjusted to a concentration of 2 × 10^7^ CFU/ml using a haemocytometer following the method described by Elad et al. [62]. For long-term storage, the bacterial cultures were kept in nutritional broth with 30 % glycerol stock at −80 °C, whereas the *T. viride* culture was kept on PDA slopes under paraffin oil at 8 °C.

### 2.3 Pathogen culture and inoculum preparation

The virulent culture of *A. solani* was procured from the Indian Type Culture Collection (ITCC), New Delhi, India (Accession number ITCC No. 3640). The fungus was cultured on potato dextrose agar (PDA) medium. The spore suspension was developed using a 10-day-old *A. solani* culture. The culture was soaked in Tween 20 and scraped out with a sterile rubber spatula. The spore suspension of the test pathogen was filtered through two layers of cheese cloth. A haemocytometer was used to determine the final spore concentration in suspension, which was adjusted to 1.5–2.0 × 10^5^ conidia per ml for inoculation in potato plants.

### 2.4 Experimental design

The experiments were established in pots under greenhouse conditions (80 % RH with 14 h of light and 10 h darkness at 27±2 °C) at the Department of Mycology and Plant Pathology, Institute of Agricultural Sciences, Banaras Hindu University, Varanasi, India. The experiment was comprised of eight treatments, i.e., *T. viride*-treated seeds, *B. subtilis*-treated seeds, *P. fluorescens*-treated seeds, *T. viride* + *B. subtilis*-treated seeds, *T. viride* + *P. fluorescens*-treated seeds, *B. subtilis* + *P. fluorescens*-treated seeds, *T. viride* + *B. subtilis* + *P. fluorescens*-treated seeds, and pathogen inoculated control. All experiments were performed thrice in triplicates, and each treatment was divided into three pots, with three tubers seeded in each pot. The tuber seeds were grown in pots (15 × 10 cm) containing 1.5 kg of mixed autoclaved soil (sandy soil, vermicompost, and farmyard manure; 2:1:1). Irrigation was provided as per the requirement. The whole experiment was repeated twice under a completely randomized design (CRD).

#### 2.4.1 Seed treatment with bioagents

The inoculum suspensions of *B. subtilis*, *P. fluorescens* (4 × 10^8^ CFU/ml), and *T. viride* (2 × 10^7^ CFU/ml) were centrifuged for 15 min at 12,000 rpm. The pellets were suspended in 100 ml of sterile distilled water. The susceptible seed tubers of the *S. tuberosum* variety ‘Kufri Bahar’ were surface sterilized for 30 s with 1 % sodium hypochlorite (NaOCl), rinsed thrice with sterile distilled water and dried under a sterile air stream. The sterilized potato seed tubers were soaked singly or in dual or triple combinations of *T. viride*, *B. subtilis*, and *P. fluorescens* bioagent suspensions. In the case of dual or triple consortia, an equal amount of suspension (v/v) was mixed and used. As an adhesive, 1 % carboxymethyl cellulose (CMC) was used. After soaking the potato seed tubers in their respective bioagent suspensions for 5–6 h, the suspension was drained, and the tubers were dried overnight on a sterile room surface. The seeds, untreated with inoculum suspensions of biocontrol agents, were used as a control.

#### 2.4.2 Measurement of morphological parameters and disease incidence

Forty-five days after sowing, three plants were randomly selected from each treatment for recording plant growth promotion activities like root and shoot height and fresh and dry weight of roots and shoots.

After four weeks of sowing, the foliar regions of the plants were sprayed with a conidial suspension of a seven-days-old culture of *A. solani* (1.5–2.0 × 10^5^ conidia per ml; 30 ml/plant) prepared in sterilized water. Potato plants were sprayed using a manual atomizer multipurpose sprayer (Chhajed Agri Private Limited, Pune, India) with an operating pressure of 60 PSI and a flow rate of 0.45 GPM, until fine water droplets were visible on the plants. The pathogen-inoculated field was immediately irrigated to maintain the soil moisture for the proper development of early blight disease. After 15 days of pathogen inoculation, the disease incidence was measured and compared to the control treatment. According to Mayee and Datar's procedure, three leaves were randomly selected from each treatment to record the disease incidence [63].

### 2.5 Sample collection for biochemical and histochemical analysis

The fresh potato leaves were collected at 24–96 h after pathogen inoculation (hapi) for biochemical analysis. Fresh plant samples from each treatment were taken, gently rinsed with running tap water, and utilized to estimate enzyme levels and changes in potato plants. The collected samples were kept at −80 °C until the studies were completed. For the histochemical analysis, leaf samples were collected at 72 hapi in an icebox and immediately used for visualization.

#### 2.5.1 Peroxidase (PO) assay

Each replicate's leaf samples (0.5 g) was homogenised in 5.0 ml of 0.1 M sodium phosphate buffer (pH 6.5). The homogenate was centrifuged for 15 min at 16,000 rpm at 4 °C, and the supernatant was utilized to calculate peroxidase enzyme activity. Then, 0.5 ml of supernatant, 1.5 ml of 0.05 M pyrogallol, and 0.5 ml of 1 % H_2_O_2_ made up the reaction mixture. The final reaction mixture was incubated at 25 ± 2 °C with the absorbance measured at 420 nm at 30 s intervals for 3 min and the peroxidase activity was represented as a change in O.D. min^−1^ g^−1^ fresh weight (FW) [64].

#### 2.5.2 Polyphenol oxidase (PPO) assay

In a pre-chilled mortar and pestle, leaf samples (0.5 g) were homogenised in 5.0 ml of 0.1 M sodium phosphate buffer (pH 6.5) and centrifuged at 16,000 rpm for 15 min at 4 °C. Then, 0.4 ml of enzyme extract, 0.4 ml of 0.01 M catechol, and 3.0 ml of 0.1 M sodium phosphate buffer were (pH 6.5) used in the process. The completed reaction mixture was incubated at 28 ± 2 °C for 5 min, and an absorbance change of 495 nm was recorded. The activity of polyphenol oxidase was measured every 30 s intervals for 3 min and reported as a change in absorbance min^−1^ g^−1^ fresh weight (FW) [65].

#### 2.5.3 Estimation of total phenolic content (TPC)

The assessment of TPC was performed according to the method of Ragazzi and Veronese [66]. Here, 100 mg of fresh leaf sample was macerated in 10 ml of 95 % ethanol followed by incubation at 70 °C in a water bath for 15 min. The homogenised tissue was centrifuged for 10 min at 13,000 rpm. The reaction mixture consisted of 1 ml of supernatant (enzyme source), 5 ml of sterile distilled water, 0.25 ml of 1 N Folin-Cicalteau's reagent (FCR) and 1 ml of sodium carbonate (5 %). The reaction mixture was vortexed and left for 15 min at room temperature. The changes in optical density of the colour developed were measured at 725 nm. The phenolic content was exhibited in μg gallic acid equivalent (GAE) g^−1^ fresh weight (FW).

#### 2.5.4 High performance liquid chromatography (HPLC) analysis of phenolic compounds in potato plant leaves

For HPLC analysis, 1 gm of fresh tissue (leaves) was harvested at 24, 48, 72, and 96 h after pathogen inoculation (hapi) according to the protocol described by Singh et al. [67]. The leaf samples were homogenised in 10 ml of 50 % methanol comprising 5 N HCl. The homogenate was kept for 24 h at room temperature and then centrifuged at 13,000 rpm for 15 min. The supernatants were collected after the completion of centrifugation, and the phenolic content was extracted using ethyl acetate. The solvent was removed with the help of a rotary evaporator (Eyela N–N series, Japan), and the residue was dissolved in HPLC-grade methanol and ready for HPLC analysis for the quantitative determination of specific phenolic compounds. The stationary phase was made of Phenomenex (Torrance, USA) C18 column (RP-Hydro, 4 μm, 250 mm × 4.6 mm) while the mobile phase used for separation of phenolic compounds was made in a gradient manner, starting from 18 % acetonitrile, moving on to 32 % at 10 min and the final concentration being 50 % at 20 min, along with 1 % glacial acetic acid. The solvent flow rate was fixed at 1.0 ml min^−1^. The composition of phenolic compounds was identified by their retention time by comparing them with their respective authentic standards at 254 nm. Analysis was performed using an HPLC system, Shimadzu model LC-10A (Japan), and data analysis was performed using Shimadzu Class VP series software. The results are illustrated in units of μg g^−1^ FW.

#### 2.5.5 Phenylalanine ammonia-lyase (PAL) activity

The approach provided by Brueske [68] was used to determine PAL activity. In this procedure, 500 mg fresh leaf samples from each treatment were pulverised in a pre-chilled mortar and pestle with 2 ml of ice-cold 100 mM sodium borate buffer (pH 8.5) containing 1.4 mM β-mercaptoethanol. The homogenised solution was centrifuged for 15 min at 16,000 rpm at 4 °C. Then, 0.2 ml of enzyme extract, 0.5 ml of borate buffer (0.2 M, pH 8.7), and 1.3 ml distilled water were added to the reaction mixture. 1 ml of 0.1 M phenylalanine (pH 8.7) was added to start the reaction, which was then incubated at 30 °C for 30 min. Then, 1 M (0.5 ml) trichloroacetic acid was added to stop the reaction process. The optical density was measured at 290 nm and the PAL activity was expressed as the amount of *trans*-Cinnamic acid (μmol TCA g^−1^ FW).

#### 2.5.6 Superoxide dismutase (SOD) activity

Fresh leaves (100 mg) from each treatment were crushed in 2 ml of extraction solution (0.1 M potassium phosphate buffer containing 0.5 mM EDTA, pH 7.5) in a pre-chilled mortar and pestle to determine the SOD activity. At 4 °C, the homogenised leaf tissue was spun for 20 min at 15,000 rpm. As a crude enzyme extract, the supernatant was employed. The reaction mixture in a test tube included 0.1 ml of supernatant (enzyme source), 200 mM methionine, 100 mM phosphate buffer (pH 7.8), 1.5 M sodium carbonate, 3.0 mM EDTA, and 2.25 mM nitroblue tetrazolium chloride. The final volume of the combination was kept at 3 ml. The reaction process was started by adding 2 mM riboflavin (0.4 ml). Each tube was exposed to a two 15-W fluorescent lamp for 20 min at 25 °C. The reaction process was stopped by switching off the light and keeping the tubes in a dark condition until the optical density was measured at 560 nm, as described by Fridovich [69]. The SOD activity was expressed as the unit g^−1^ fresh weight (FW).

#### 2.5.7 β-1,3-glucanase activity

The activity of β-1,3-glucanase was measured using the method of Pan et al. [70]. This method involved macerating 100 mg of fresh leaf tissue in 2 ml of 0.05 M sodium borate buffer (pH 5.0) and then centrifuging the macerated leaf sample at 16,000 rpm for 15 min at 4 °C. The resulting supernatant that resulted was used as an enzyme source. A reaction combination of 0.3 ml 1 M sodium acetate buffer (pH 5.3), 0.25 ml supernatant (enzyme source), and 0.5 ml 4 % laminarin was added to a test tube. The reaction mixture was incubated at 40 °C for 60 min and terminated by adding 0.375 ml of 3,5-dinitrosalicylic acid, which was then heated for 5 min in a boiling water bath. The changes in optical density were recorded spectrophotometrically at 500 nm and the enzyme activity was expressed as μg glucose released min^−1^ g^−1^ fresh weight (FW).

#### 2.5.8 Chitinase

The chitinase enzyme activity was determined according to Boller and Munch [71] with slight modifications and colloidal chitin was prepared by acetylation of glycol chitosan using the protocol described by Trudel and Asselin [72]. Fresh leaf samples (0.1 g) were homogenised in 2.0 ml sodium citrate buffer (pH 5.0) and centrifuged at 14,000 rpm for 20 min at 4 °C. The reaction mixture containing 0.1 ml enzyme extract and 0.1 ml colloidal chitin was incubated at normal room temperature for 2 h, and the reaction process was stopped by centrifugation at 10,000 rpm for 3 min at 4 °C. 0.3 ml supernatant (enzyme source) was taken in sterilized glass tube and was added into 0.03 ml 1 M potassium phosphate buffer (pH 7.1) and then incubated with 0.2 ml desalted snail helicase. Finally, the reaction mixture was incubated with 0.2 ml dimethylaminobenzaldehyde (Sigma-Aldrich) for 30 min at room temperature, and the absorbance was measured at a wavelength of 585 nm. The chitinase enzyme activity was defined as μmols GlcNAc released min^−1^ g^−1^ FW, and their *N*-acetylglucosamine (GlcNAc) was used as a standard.

#### 2.5.9 Catalase (CAT) assay

With the help of a pre-chilled mortar and pestle, 0.2 mg fresh potato leaf samples were collected from inoculated and non-inoculated plants and homogenised in 50 mM Tris HCl buffer (pH 8.0) containing 0.5 mM EDTA, 2 % polyvinylpyrrolidone (w/v), and 0.5 % Triton X100 (v/v). At 4 °C, the homogenate was centrifuged for 10 min at 16,000 rpm. The enzyme source was the final supernatant. Phosphate buffer (25 mM, pH 7.0), 10 mM H_2_O_2_, and 0.25 ml supernatant were used in the process. The O_2_ produced by enzymatic dissociation of H_2_O_2_ was measured in the dark for 1 min to estimate CAT activity. At a wavelength of 240 nm, activity was measured and expressed as μM H_2_O_2_ oxidized min^−1^ g^−1^ FW [73].

### 2.6 Histochemical analysis

#### 2.6.1 In-situ hydrogen peroxide (H_2_O_2_) localization

Histochemical detection of H_2_O_2_ was confirmed by using the 3,3′ -diaminobenzidine (DAB; HiMedia, India) staining method as described by Sakamoto et al. [74]. Fresh potato leaves were randomly collected at 72 hapi from each treatment and used for the detection of H_2_O_2_. The treated and untreated leaves were dipped in 2 ml of DAB solution and adjusted to pH 3.8 with KOH. The leaves were kept in dark conditions for 3 h. After incubation, leaves were bleached in a bleaching solution containing acetic acid-glycerol- ethanol (1:1:3, v/v/v), and then leaves were incubated at 100 °C until the chlorophyll pigment was completely removed. The histochemical appearance of the reddish-brown color formation in the leaves indicates the H_2_O_2_ deposition that was observed. Photographs were taken under a light microscope.

### 2.7 Statistical analysis

IBM SPSS Version 20 was used for the analysis. The provided values from several experiments were the average of three replications, and the standard deviation (SD) of the mean was indicated. The current data was examined statistically using one-way analysis of variance (ANOVA), and treatment mean values were compared using Duncan's multiple range tests at the 0.05 significance level. R studio software, version 1.4.1717, was used to perform principal component analysis (PCA) to explore the correlations between the eight different treatments. On the other hand, using the means of all data for the study treatments, similarities and variation in all response treatments were expressed as a heatmap and two-way cluster hierarchical analysis.

## 3 Results

### 3.1 Effect of the microbial consortium on plant growth promotion activities and the early blight disease response

**Fig. 1:**
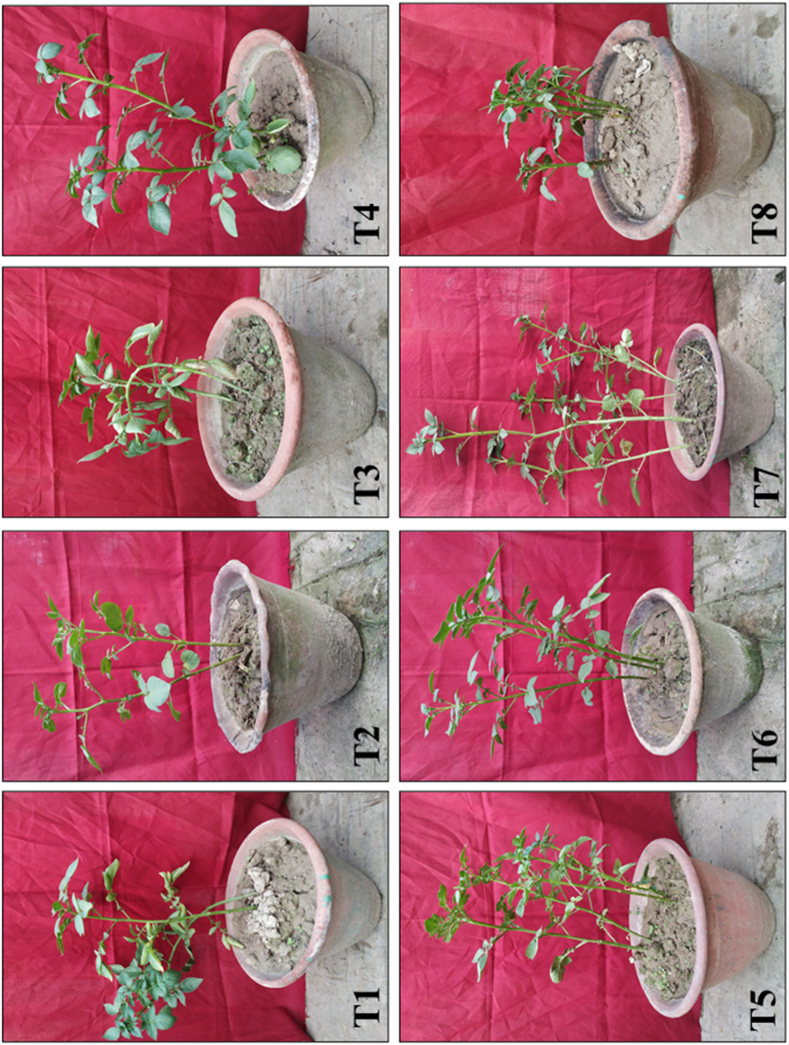
Effect of individual or consortia of microbial bioagents on the growth promotion activities of potato plants under greenhouse conditions. (T1) *Trichoderma viride*; (T2) *Bacillus subtilis*; (T3) *Pseudomonas fluorescens*; (T4) *Trichoderma viride* + *Bacillus subtilis*; (T5) *Trichoderma viride* + *Pseudomonas fluorescens*; (T6) *Bacillus subtilis* + *Pseudomonas fluorescens*; (T7) *Trichoderma viride + Bacillus subtilis* + *Pseudomonas fluorescens*; (T8) Untreated pathogen challenged control.

**Fig. 2:**
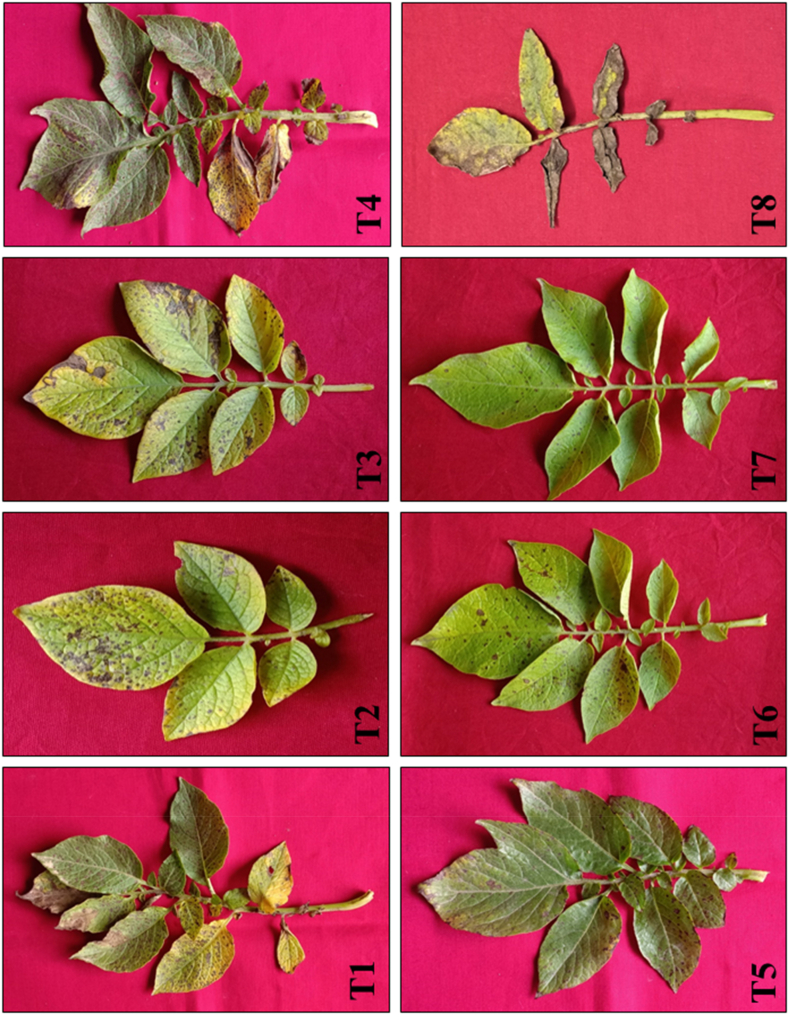
Effect of single or double or triple species microbial consortium treatments on disease incidence of potato plants after challenged inoculation with *A. solani* under greenhouse conditions. (T1) *Trichoderma viride*; (T2) *Bacillus subtilis*; (T3) *Pseudomonas fluorescens*; (T4) *Trichoderma viride* + *Bacillus subtilis*; (T5) *Trichoderma viride* + *Pseudomonas fluorescens*; (T6) *Bacillus subtilis* + *Pseudomonas fluorescens*; (T7) *Trichoderma viride* + *Bacillus subtilis* + *Pseudomonas fluorescens*; (T8) Control.

**Fig. 3:**
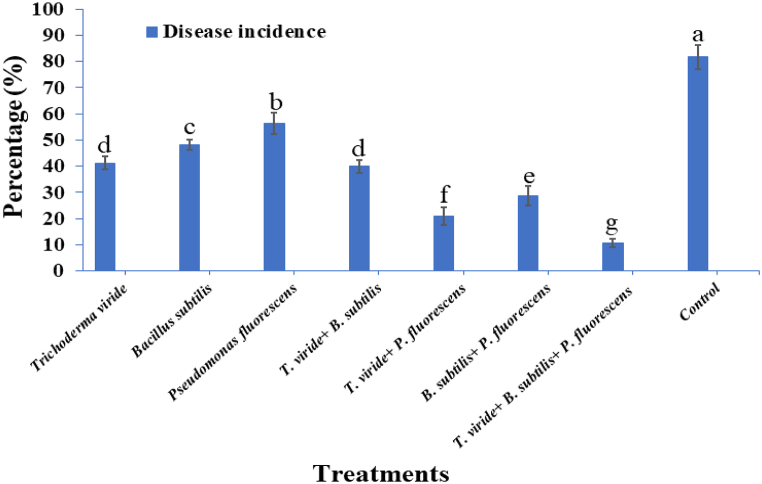
Early blight incidence in potato plants treated with *T. viride*, *B. subtilis*, and *P. fluorescens* either individually or in mixture and challenged with *A. solani* under greenhouse conditions. Each value is expressed as the average of three replications, where vertical bars designate the standard deviations of the mean. Different alphabetical letters designate significant differences among treatments using Duncan's multiple range test at p ≤ 0.05.

Potato tubers primed with the microbial consortium either singly or in combination had significantly increased plant growth in terms of shoot length, root length, and fresh and dry weight of shoots and roots in comparison to *A. solani*-challenged control plants (Fig. 1). However, among all the treatments, three-microbe consortium-treated tubers showed significantly higher potato plant growth in terms of shoot length (34.5 cm), root length (16.36 cm), fresh weight of shoot (20.86 g), fresh weight of root (3.95 g), and dry weight of shoot and root (1.98 and 1.03 g), respectively, compared to singly or double bioagents-treated plants and pathogen-inoculated control plants. However, individual bioagent treated tubers exhibited the lowest plant growth promotion activities when compared with consortium-treated plants. The pathogen-inoculated control plants showed the least plant growth promotion (Table 1). The early blight disease response results indicated that the pathogen inoculated control plants expressed maximum disease incidence and resulted in concentric rings developed on the leaves. The response of early blight development under single or consortium-treated treatments is shown in Fig. 2. Among all the treatments, the three-species microbe consortium showed the lowest disease incidence compared to single or double microbe-treated plants. The minimum disease incidence was observed in plants treated with the three-microbe consortium of *T. viride* + *B. subtilis* + *P. fluorescens* (10.61 %), followed by *T. viride* + *P. fluorescens* (20.93 %) treated plants in comparison to pathogen inoculated control plants. On the other hand, the highest early blight incidence was recorded in the pathogen-inoculated control (81.63 %), followed by *P. fluorescens* (56.26 %) and *B. subtilis* (48.34 %) treated plants (Fig. 3).

**Table 1:** Effect of the microbial consortium on the growth promoting activities of potato plants under greenhouse conditions.

Treatments	Shoot length (cm)	Root length (cm)	Shoot fresh weight (g)	Root fresh weight (g)	Shoot dry weight (g)	Root dry weight (g)
*Trichoderma viride*	20.56 ± 1.11^e^	11.96 ± 0.40^cd^	10.33 ± 1.92^cd^	2.85 ± 0.08^cde^	1.43 ± 0.09^cd^	0.59 ± 0.03^c^
*Bacillus subtilis*	23.76 ± 0.60^d^	10.46 ± 1.00^d^	11.06 ± 1.55^c^	2.69 ± 0.14^de^	1.45 ± 0.06^cd^	0.47 ± 0.03^d^
*Pseudomonas fluorescens*	17.6 ± 1.71^f^	9.93 ± 0.57^de^	9.26 ± 0.93^d^	2.54 ± 0.11^ef^	1.29 ± 0.03^d^	0.39 ± 0.06^de^
*T. viride* + *B. subtilis*	26.86 ± 1.26^c^	14.73 ± 1.57^ab^	12.53 ± 1.89^b^	3.44 ± 0.26^b^	1.55 ± 0.11^c^	0.90 ± 0.06^b^
*T. viride* + *P. fluorescens*	31.06 ± 1.82^b^	13.3 ± 1.06^bc^	18.4 ± 0.80^a^	3.14 ± 0.12^bc^	1.90 ± 0.05^ab^	0.86 ± 0.03^b^
*B. subtilis* + *P. fluorescens*	28.06 ± 1.02^c^	12.06 ± 1.43^cd^	14.2 ± 1.68^b^	2.99 ± 0.02^cd^	1.76 ± 0.05^b^	0.66 ± 0.06^c^
*T. viride* + *B. subtilis* + *P. fluorescens*	34.5 ± 1.55^a^	16.36 ± 0.55^a^	20.86 ± 2.68^a^	3.95 ± 0.19^a^	1.98 ± 0.09^a^	1.03 ± 0.05^a^
Control	12.1 ± 2.27^g^	8.03 ± 0.60^e^	8.53 ± 0.73^e^	2.08 ± 0.12^f^	1.1 ± 0.06^e^	0.31 ± 0.08^e^
CD (0.05 %)	2.57	2.24	2.42	0.34	0.17	0.09

The results are the average of three replications and ± represent standard deviations of the mean. Different alphabetical letters on the superscript indicate significant differences between the treatments according to Duncan's multiple range test at p ≤ 0.05.

### 3.2 Effect of the microbial consortium on defense enzyme activity

#### 3.2.1 Peroxidase (PO) activity

**Fig. 4:**
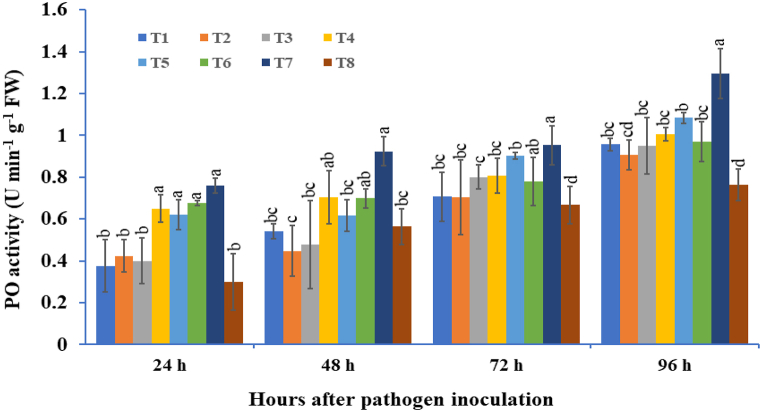
The effect of three bioagents (Tv = *Trichoderma viride*, Bs = *Bacillus subtilis* and Pf = *Pseudomonas fluorescens*) used either singly or in combination on peroxidase (PO) activity at different time intervals in potato plants challenged with *Alternaria solani*. The results are expressed as the average of three replications, and the vertical bars show the standard deviations of the means. The different alphabetical letters show significant differences among treatments by using Duncan's multiple range test at p ≤ 0.05. (T1) Tv; (T2) Bs; (T3) Pf; (T4) Tv + Bs; (T5) Tv + Pf; (T6) Bs + Pf; (T7) Tv + Bs + Pf; (T8) Control.

The potato tubers primed with three bioagents namely *T. viride*, *B. subtilis*, and *P. fluorescens,* were used either individually or in combination to induce PO activity from 24 to 96 hapi when compared with pathogen inoculated BCA untreated control plants (Fig. 4). Within all the treatments, the consortium of *T. viride* + *B. subtilis* + *P. fluorescens* primed potato tubers showed strongly enhanced PO activity after *A. solani* inoculation. The highest PO activity was recorded in the consortium of *T. viride* + *B. subtilis* + *P. fluorescens* treated plants, followed by *T. viride* + *P. fluorescens* treated plants and the lowest PO activity was recorded in the untreatedchallenged plants. In the peroxidase enzymatic assay, potato tubers bioprimed with mixture of three bioagents was showed maximum activity (1.30 U min^−1^ g^−1^ FW) followed by a consortium of *T. viride* + *P. fluorescens* (1.05 U min^−1^ g^−1^ FW) treated potato tubers at 96 hapi. The peroxidase activity was recorded to be approximately 1.71 times higher in plants treated with the consortium of *T. viride* + *B. subtilis* + *P. fluorescens* followed by 1.72 times higher in *T. viride* + *P. fluorescens* treated plants compared to the control plants at 96 hapi. From Fig. 4 it is clear that in the three bioagents consortium treated plants; the PO activity started to increase at 24 hapi and reached highest at 96 hapi.

#### 3.2.2 Polyphenol oxidase (PPO) activity

**Fig. 5:**
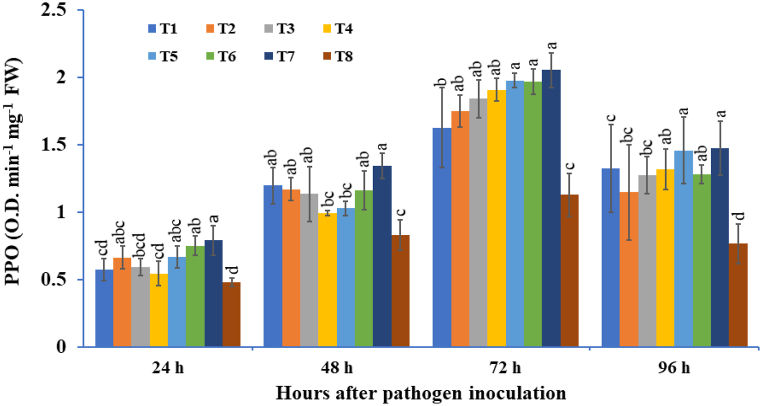
The effect of three bioagents (Tv = *Trichoderma viride*, Bs = *Bacillus subtilis*, and Pf = *Pseudomonas fluorescens*) used either singly or in combination on polyphenol oxidase (PPO) activity at different time intervals in potato plants challenged with *Alternaria solani*. The results are expressed as the average of three replications, and the vertical bars show the standard deviations of the means. The different alphabetical letters show significant differences among treatments by using Duncan's multiple range test at p ≤ 0.05. (T1) Tv; (T2) Bs; (T3) Pf; (T4) Tv + Bs; (T5) Tv + Pf; (T6) Bs + Pf; (T7) Tv + Bs + Pf; (T8) Control.

The PPO activity of potato tubers inoculated with three bioagents used either singly or in double or triple combinations of microorganisms, that is, *T. viride*, *B. subtilis*, and *P. fluorescens* under pathogen-challenged conditions was studied and compared with *A. solani*-challenged control samples (Fig. 5). In potato tubers treated with bioagents, the PPO activity increased from 24 hapi and reached a maximum at 72 hapi, and thereafter, it declined. All the bioagent treated potato tubers expressed significantly higher PPO activity compared to pathogen-inoculated control plants. In these results, higher activity of PPO was recorded in the plants treated with a consortium of *T. viride* + *B. subtilis* + *P. fluorescens* (2.05 O.D. min^−1^ mg^−1^ FW), followed by *T. viride* + *P. fluorescens* (1.97 O.D. min^−1^ mg^−1^ FW) treated potato tubers at 72 hapi. Among bioagent treated tubers, minimum PPO activity was observed in individually *T. viride* (1.62 O.D. min^−1^ mg^−1^ FW)-treated tubers at 72 hapi, for the pathogen-inoculated control. The three-microbe consortium showed 1.83 times higher PPO activity when compared with the pathogen-inoculated control.

#### 3.2.3 Phenylalanine ammonia-lyase (PAL) activity

**Fig. 6:**
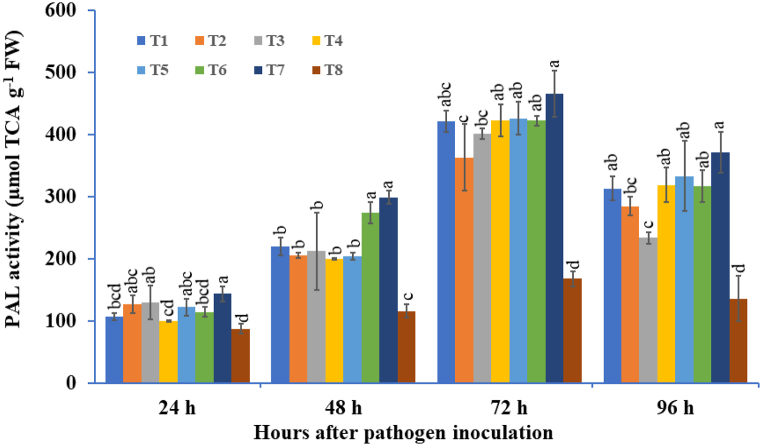
The effect of three bioagents (Tv = *Trichoderma viride*, Bs = *Bacillus subtilis*, and Pf = *Pseudomonas fluorescens*) used either singly or in combination on phenylalanine ammonia-lyase (PAL) activity at different time intervals in potato plants challenged with *Alternaria solani*. The results are expressed as the average of three replications, and the vertical bars show the standard deviations of the means. The different alphabetical letters show significant differences among treatments by using Duncan's multiple range test at p ≤ 0.05. (T1) Tv; (T2) Bs; (T3) Pf; (T4) Tv + Bs; (T5) Tv + Pf; (T6) Bs + Pf; (T7) Tv + Bs + Pf; (T8) Control.

Potato tubers treated with plant growth-promoting bacteria (PGPB) and plant growth-promoting fungi (PGPF) under pathogen-inoculated conditions were observed to significantly induce PAL activity (Fig. 6). Under the influence of bioagent treatments, the PAL activity significantly increased at 24 hapi and reached its maximum at 72 hapi, followed by a decline. The three species bioagent consortium, followed by the two species bioagent consortium-treated tubers, showed higher PAL activity at 72 hapi compared to individually treated bioagents and pathogen-inoculated control plants. The maximum activity of PAL was recorded in the treatments treated with a three-species microbial consortium to be approximately 2.77 times higher, followed by two species microbe consortia, that is, *T. viride* + *P. fluorescence*, which represents 2.53 times higher at 72 hapi in comparison to their corresponding pathogen-inoculated control and was significantly higher when compared to all other bioagent treated treatments.

### 3.3 Effect of the microbial consortium on antioxidant enzyme activity

#### 3.3.1 Superoxide dismutase (SOD) activity

**Fig. 7:**
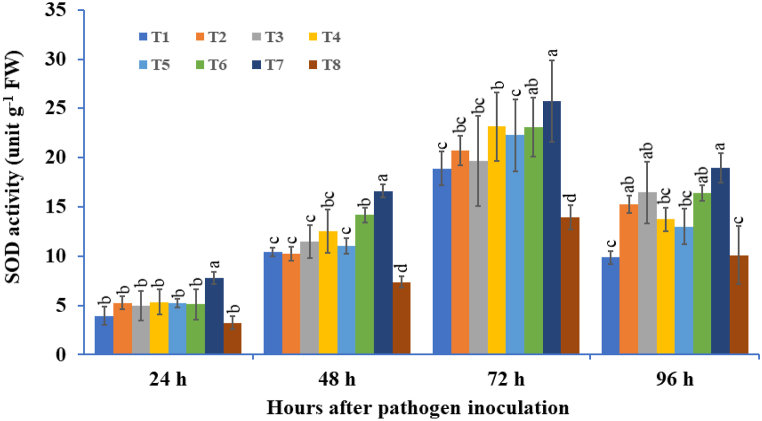
The effect of three bioagents (Tv = *Trichoderma viride*, Bs = *Bacillus subtilis*, and Pf = *Pseudomonas fluorescens*) used either singly or in combination on superoxide dismutase (SOD) activity at different time intervals in potato plants challenged with *Alternaria solani*. The results are expressed as the average of three replications, and the vertical bars show the standard deviations of the means. The different alphabetical letters show significant differences among treatments by using Duncan's multiple range test at p ≤ 0.05. (T1) Tv; (T2) Bs; (T3) Pf; (T4) Tv + Bs; (T5) Tv + Pf; (T6) Bs + Pf; (T7) Tv + Bs + Pf; (T8) Control.

The individual or combination of microbe-treated plants inoculated with pathogens showed a successive induction of SOD activity constantly up to 72 hapi in all bioagent-treated tubers and thereafter a decline at 96 hapi (Fig. 7). The maximum levels of SOD were observed when *T. viride* + *B. subtilis* + *P. fluorescens* were applied together compared with the rest of the treatments. SOD activity was higher in three microbe consortia (25.75-unit g^−1^ FW) followed by *T. viride* + *B. subtilis* (23.15 -unit g^−1^ FW) treated tubers as compared to the pathogen-inoculated control at 72 hapi. The minimum SOD activity was recorded in singly *T. viride*-treated tubers under pathogen inoculation at 72 hapi, followed by pathogen-inoculated control plants. The maximum activity was recorded to be approximately 1.84 times higher in treatments inoculated with the three-species microbial consortium, followed by 1.65 times higher in *T. viride* + *B. subtilis* consortium-treated tubers compared to the pathogen-inoculated control at 72 hapi (Fig. 7).

#### 3.3.2 Catalase (CAT) activity

**Fig. 8:**
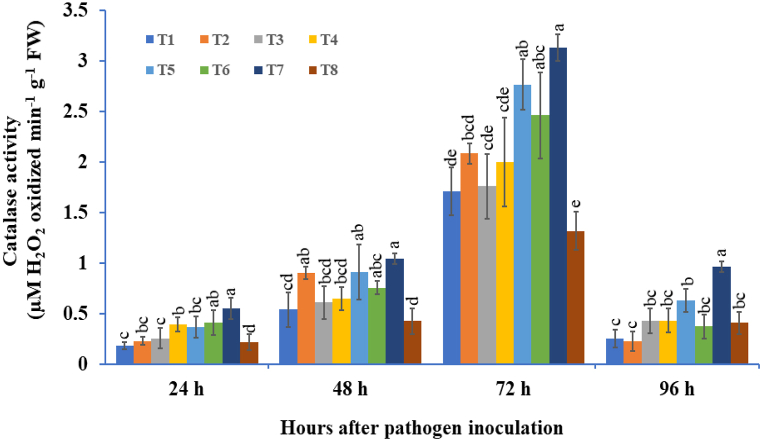
The effect of three bioagents (Tv = *Trichoderma viride*, Bs = *Bacillus subtilis*, and Pf = *Pseudomonas fluorescens*) used either singly or in combination on the catalase (CAT) activity at different time intervals in potato plants challenged with *Alternaria solani*. The results are expressed as the average of three replications, and the vertical bars show the standard deviations of the means. The different alphabetical letters show significant differences among treatments by using Duncan's multiple range test at p ≤ 0.05. (T1) Tv; (T2) Bs; (T3) Pf; (T4) Tv + Bs; (T5) Tv + Pf; (T6) Bs + Pf; (T7) Tv + Bs + Pf; (T8) Control.

In the present results, the potato tubers treated with single or double or triple consortia of bioagents under challenge with *A. solani* showed successive augmentation of levels of CAT, which increased from 24 hapi, reached a maximum at 72 hapi, and thereafter declined (Fig. 8). Among all the treatments, the microbial consortium of three-species treated tubers strongly induced CAT activity after *A. solani* inoculation at 72 hapi, followed by *B. subtilis* + *P. fluorescens* treated tubers. In these results, the CAT activity was highest in the *T. viride* + *B. subtilis* + *P. fluorescens* consortium (3.13 μM H_2_O_2_ oxidized min^−1^ g^−1^ FW), followed by *T. viride* + *B. subtilis* (2.76 μM H_2_O_2_ oxidized min^−1^ g^−1^ FW) treated tubers at 72 hapi. The lowest CAT activity was observed singly *T. viride*-treated tubers, followed by the pathogen-inoculated control (Fig. 8).

### 3.4 Effect of the microbial consortium on PR proteins

#### 3.4.1 Chitinase activity

**Fig. 9:**
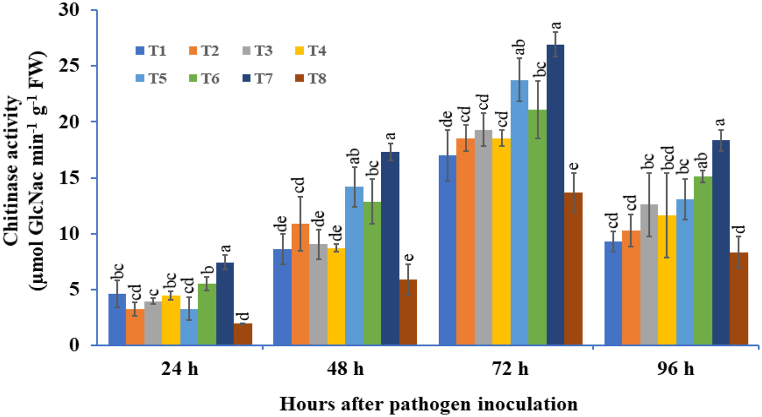
The effect of three bioagents (Tv = *Trichoderma viride*, Bs = *Bacillus subtilis*, and Pf = *Pseudomonas fluorescens*) used either singly or in combination on chitinase activity at different time intervals in potato plants challenged with *Alternaria solani*. The results are expressed as the average of three replications, and the vertical bars show the standard deviations of the means. The different alphabetical letters show significant differences among treatments by using Duncan's multiple range test at p ≤ 0.05. (T1) Tv; (T2) Bs; (T3) Pf; (T4) Tv + Bs; (T5) Tv + Pf; (T6) Bs + Pf; (T7) Tv + Bs + Pf; (T8) Control.

The potato tubers primed with microbial BCAs used either individually or combined under *A. solani*-challenged conditions significantly enhanced the chitinase activity at different time intervals (Fig. 9). The highest chitinase activity was recorded in triple-microbial consortium-treated potato tubers, followed by the double-species microbial consortium, that is, *T. viride* + *P. fluorescens* at 72 hapi. The minimum chitinase activity was observed in the pathogen-inoculated control (13.68 μmol GlcNac min^−1^ g^−1^ FW), followed by individually *T. viride*-treated tubers (17.01 μmol GlcNac min^−1^ g^−1^ FW). The highest activity was recorded in *T. viride* + *B. subtilis* + *P. fluorescens*-treated plants (1.96 times higher), followed by *T. viride* + *P. fluorescens* (1.73 times higher) as compared to the pathogen-inoculated control at 72 hapi, as shown in Fig. 9. In bioagent treated tubers, the chitinase activity started to increase at 24 hapi and reached its maximum at 72 hapi thereafter, a declining trend was observed.

#### 3.4.2 β-1,3-glucanase activity

**Fig. 10:**
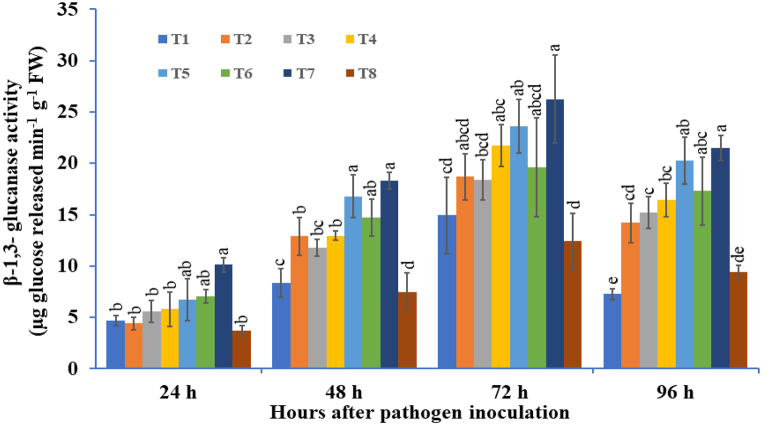
The effect of three bioagents (Tv = *Trichoderma viride*, Bs = *Bacillus subtilis*, and Pf = *Pseudomonas fluorescens*) used either singly or in combination on β-1,3-glucanase activity at different time intervals in potato plants challenged with *Alternaria solani*. The results are expressed as the average of three replications, and the vertical bars show the standard deviations of the means. The different alphabetical letters show significant differences among treatments by using Duncan's multiple range test at p ≤ 0.05. (T1) Tv; (T2) Bs; (T3) Pf; (T4) Tv + Bs; (T5) Tv + Pf; (T6) Bs + Pf; (T7) Tv + Bs + Pf; (T8) Control.

In our results, the potato tubers primed with bioagents under *A. solani* challengedconditions expressed an increase in the levels of β-1,3-glucanase at different time intervals (Fig. 10). The three-microbe consortium treatment significantly enhanced the activity of β-1,3-glucanase as compared to double or single bioagent-treated tubers. The lowest activity was recorded in pathogen-inoculated control plants, followed by individually treated *T. viride* tubers. The significant induction in β-1,3-glucanase activity was recorded at 24 hapi, followed by gradual enhancement at 72 hapi, and decreased thereafter. At 72 hapi, the highest β-1,3-glucanase activity was recorded in the three-species microbial consortium, that is, *T. viride* + *B. subtilis* + *P. fluorescens*-treated tubers (26.24 μg glucose released min^−1^ g^−1^ FW), followed by *T. viride* + *P. fluorescens* (23.60 μg glucose released min^−1^ g^−1^ FW). The three-microbe mixture recorded 2.10 times higher activity, followed by a double mixture of *T. viride* + *P. fluorescens* bioagent 1.89 times higher when compared to the pathogen-inoculated control at 72 hapi (Fig. 10). Thereafter, a decreasing trend was observed at 96 hapi in all the treatments.

### 3.5 Effect of microbial consortia on TPC in leaves

**Fig. 11:**
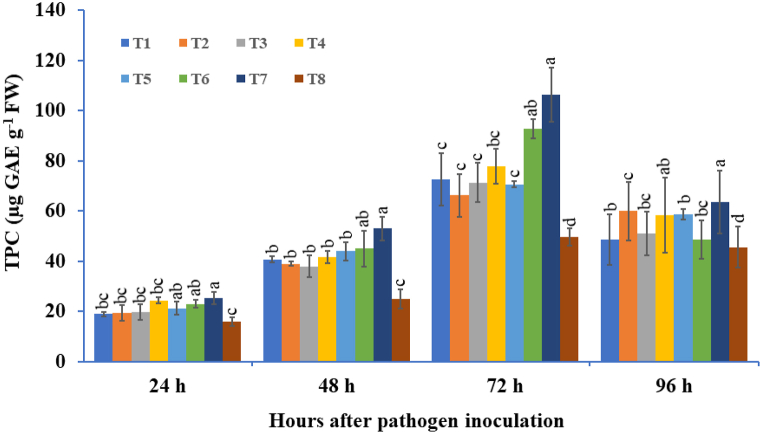
The effect of three bioagents (Tv = *Trichoderma viride*, Bs = *Bacillus subtilis*, and Pf = *Pseudomonas fluorescens*) used either singly or in combination on the total phenolic content (TPC) at different time intervals in potato plants challenged with *Alternaria solani*. The results are expressed as the average of three replications, and the vertical bars show the standard deviations of the means. The different alphabetical letters show significant differences among treatments by using Duncan's multiple range test at p ≤ 0.05. (T1) Tv; (T2) Bs; (T3) Pf; (T4) Tv + Bs; (T5) Tv + Pf; (T6) Bs + Pf; (T7) Tv + Bs + Pf; (T8) Control.

The highest levels of total phenolic content (TPC) were found in the microbial consortium-treated treatments under pathogen-challenged conditions at different time intervals (Fig. 11). The TPC content in the individual or combined mixture of bioagent-treated treatments started to increase at 24 hapi, followed by a gradual increment up to 72 hapi, and thereafter a decreasing trend was observed. The phenolic content was found to be significantly higher in three-microbe mixture-treated treatments followed by a double mixture of microbes and individually *T. viride*-treated treatments compared to the *A. solani*-inoculated control. The potato tubers bioprimed with a mixture of three-microbes and challenged with *A. solani* showed maximum TPC content, that is, 2.14 times higher, followed by the mixture of two-microbes, *B. subtilis* + *P. fluorescens*, viz., 1.86 times higher than the corresponding pathogen-challenged control tubers at 72 hapi. Among individual microbe-treated treatments, *T. viride*-treated tubers challenged with *A. solani* showed significantly higher TPC content than the pathogen-inoculated control treatment (Fig. 11).

### 3.6 Effect of the consortium on free phenolic compounds in potato leaves

**Fig. 12:**
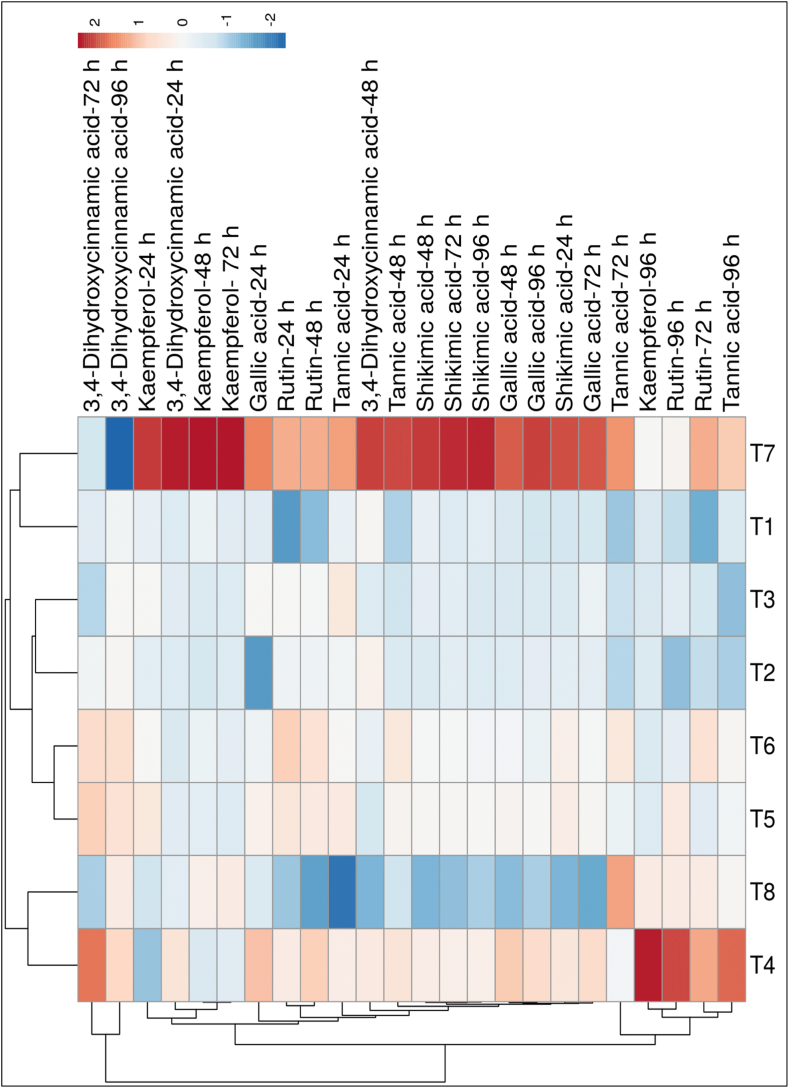
Clustering and heatmap analysis of the free phenolic profile. The rows express the individual phenolic compounds, and the columns indicated the treatments (T1) Tv; (T2) Bs; (T3) Pf; (T4) Tv + Bs; (T5) Tv + Pf; (T6) Bs + Pf; (T7) Tv + Bs + Pf; (T8) Control. Lower numerical values are blue colour, whereas higher numerical values are red. The map was generated by using the ClustVis website, https://biit.cs.ut.ee/clustvis/. The results are expressed as the average of three replications. (For interpretation of the references to colour in this figure legend, the reader is referred to the Web version of this article.)

The significant variation in free phenolic accumulation was recorded in potato tubers treated with BCAs either alone or in consortium challenges with *A. solani* at different time intervals, using HPLC (Table 2A, Table 2B, Table 2C, Table 2DD). In this experiment, the quantitative analysis of six potential phenolic compounds, such as shikimic acid, gallic acid, kaempferol, rutin, 3, 4-dihydroxycinnamic acid, and tannic acid, was identified; details are given in Fig. 12. The results showed that the values of phenolic compounds were significantly higher in single, dual, or triple microbe consortium-treated tubers than in pathogen-inoculated control potato tubers. The significant induction in all phenolic compounds increased from 24 hapi and reached a higher level at 72 hapi; thereafter, it declined. Out of all the free phenolics analyzed, shikimic acid (1342.77 ± 13.91 μg g^−1^ FW) was found to be the highest, followed by rutin (133.48 ± 2.79 μg g^−1^ FW) and tannic acid (128.57 ± 0.96 μg g^−1^ FW) at 72 hapi. Among all the treatments at different time intervals, the *T. viride* + *B. subtilis* + *P. fluorescens* pathogen challenged tubers strongly stimulated the phenolic compounds compared to single or dual consortium-treated tubers, and an untreated control was found at 72 hapi. The treatment with highest potential was the triple-species microbial consortium, where shikimic acid was 3.47 times higher, gallic acid 2.81 times, kaempferol 1.72 times, rutin 1.10 times, 3,4-dihydroxycinnamic acid 1.30 times, and tannic acid 1.02 times higher at 72 hapi when compared to the control, where the tubers were inoculated only with *A. solani* (Table 2C). The results indicated that the triple-microbe species consortium-treated tubers challenged with pathogen showed significantly higher phenolic compounds, followed by the dual consortium and single species of biocontrol agents (Table 2A, Table 2B, Table 2C, Table 2DD).

**Table 2A:** Effect of microbes in either individual or consortium treatments on the phenolic content (μg g^−1^ FW) in the leaves of potato plants at 24 h after pathogen inoculation.

Treatments/Phenolics	T1	T2	T3	T4	T5	T6	T7	T8
Shikimic acid	432.51 ± 7.19^e^	463.61 ± 5.62^d^	444.42 ± 2.92^e^	566.00 ± 2.46^b^	539.60 ± 17.26^c^	536.14 ± 11.14^c^	739.85 ± 6.03^a^	348.75 ± 5.98^f^
Gallic acid	43.52 ± 1.93^de^	32.79 ± 4.27^f^	47.64 ± 2.22^cd^	56.36 ± 2.92^b^	48.84 ± 2.42^c^	46.28 ± 1.77^cde^	61.08 ± 3.67^a^	42.76 ± 2.85^e^
Kaempferol	0.54 ± 0.03^bc^	0.53 ± 0.02^bcd^	0.57 ± 0.03^bc^	0.46 ± 0.03^d^	0.60 ± 0.04^b^	0.57 ± 0.03^bc^	0.75 ± 0.10^a^	0.50 ± 0.03^cd^
Rutin	55.21 ± 3.39^e^	83.10 ± 4.12^c^	86.48 ± 3.73^bc^	91.52 ± 4.73^b^	93.86 ± 4.83^b^	101.33 ± 4.75^a^	107.42 ± 5.76^a^	65.31 ± 2.78^d^
3,4-Dihydrocinnamic acid	3.56 ± 0.04^c^	3.55 ± 0.02^c^	3.62 ± 0.03^c^	4.63 ± 0.07^b^	3.61 ± 0.03^c^	3.47 ± 0.35^c^	6.63 ± 0.38^a^	3.65 ± 0.29^c^
Tannic acid	54.45 ± 0.39^d^	57.10 ± 3.43^cd^	65.54 ± 3.41^b^	63.29 ± 1.84^b^	64.53 ± 1.31^b^	59.38 ± 2.80^c^	80.71 ± 3.63^a^	24.51 ± 0.78^e^

The results are expressed as the average of three replications and ± represent standard deviations of the mean. Different alphabetical letters on the superscript indicate significant differences between the treatments according to Duncan's multiple range test at p ≤ 0.05. (T1) Tv; (T2) Bs; (T3) Pf; (T4) Tv + Bs; (T5) Tv + Pf; (T6) Bs + Pf; (T7) Tv + Bs + Pf; (T8) Control.

**Table 2B:** Effect of microbes in either individual or consortium treatments on the phenolic content (μg g^−1^ FW) in the leaves of potato plants at 48 h after pathogen inoculation.

Treatments/Phenolics	T1	T2	T3	T4	T5	T6	T7	T8
Shikimic acid	550.96 ± 6.36^e^	507.51 ± 4.52^f^	543.03 ± 3.78^e^	651.78 ± 11.68^b^	613.43 ± 8.80^c^	601.22 ± 3.34^d^	985.26 ± 2.17^a^	356.67 ± 6.36^g^
Gallic acid	51.78 ± 3.81^d^	52.90 ± 1.99^d^	51.75 ± 3.24^d^	78.66 ± 3.99^b^	63.75 ± 2.21^c^	60.40 ± 0.94^c^	95.11 ± 2.22^a^	39.18 ± 0.82^e^
Kaempferol	0.57 ± 0.04^c^	0.50 ± 0.02^d^	0.52 ± 0.03^cd^	0.51 ± 0.03^d^	0.54 ± 0.02^cd^	0.57 ± 0.02^c^	1.00 ± 0.06^a^	0.64 ± 0.03^b^
Rutin	85.84 ± 3.03^e^	100.45 ± 2.95^d^	102.11 ± 3.40^d^	113.44 ± 4.07^b^	107.18 ± 0.67^c^	109.47 ± 0.90^bc^	118.15 ± 1.84^a^	81.57 ± 0.60^e^
3,4-Dihydrocinnamic acid	4.31 ± 0.06^bc^	4.35 ± 0.16^bc^	4.03 ± 0.07^d^	4.45 ± 0.22^b^	3.94 ± 0.11^d^	4.14 ± 0.09^cd^	5.28 ± 0.05^a^	3.57 ± 0.23^e^
Tannic acid	62.25 ± 3.32^e^	68.10 ± 1.54^d^	65.42 ± 3.52^de^	82.26 ± 2.01^b^	76.89 ± 2.75^c^	80.77 ± 1.48^bc^	101.55 ± 2.12^a^	65.27 ± 1.83^de^

The results are expressed as the average of three replications and ± represent standard deviations of the mean. Different alphabetical letters on the superscript indicate significant differences between the treatments according to Duncan's multiple range test at p ≤ 0.05. (T1) Tv; (T2) Bs; (T3) Pf; (T4) Tv + Bs; (T5) Tv + Pf; (T6) Bs + Pf; (T7) Tv + Bs + Pf; (T8) Control.

**Table 2C:** Effect of microbes in either individual or consortium treatments on the phenolic content (μg g^−1^ FW) in the leaves of potato plants at 72 h after pathogen inoculation.

Treatments/Phenolics	T1	T2	T3	T4	T5	T6	T7	T8
Shikimic acid	591.50 ± 8.14^bc^	620.13 ± 6.02^b^	614.30 ± 5.85^b^	789.80 ± 7.99^ab^	751.69 ± 5.29^ab^	731.86 ± 2.24^ab^	1342.77 ± 13.91^a^	386.28 ± 6.92^c^
Gallic acid	55.85 ± 4.16^e^	61.12 ± 1.67^d^	63.66 ± 3.00^d^	81.66 ± 2.01^b^	69.60 ± 1.13^c^	67.94 ± 1.29^c^	106.14 ± 0.36^a^	37.67 ± 0.87^f^
Kaempferol	0.56 ± 0.04^c^	0.54 ± 0.03^c^	0.54 ± 0.04^c^	0.56 ± 0.03^c^	0.54 ± 0.02^c^	0.58 ± 0.02^c^	1.29 ± 0.06^a^	0.75 ± 0.04^b^
Rutin	94.93 ± 2.28^e^	103.27 ± 1.58^d^	106.82 ± 3.34^c^	132.81 ± 0.64^a^	109.08 ± 2.20^c^	123.81 ± 2.18^b^	133.48 ± 2.79^a^	120.43 ± 0.70^b^
3,4-Dihydrocinnamic acid	4.69 ± 0.17^de^	4.86 ± 0.13^cd^	4.44 ± 0.11^de^	4.56 ± 0.30^de^	5.34 ± 0.07^ab^	5.26 ± 0.08^bc^	5.75 ± 0.30^a^	4.40 ± 0.50^e^
Tannic acid	73.59 ± 4.36^e^	77.24 ± 1.74^d^	80.17 ± 1.08^d^	96.85 ± 0.53^c^	93.57 ± 0.92^c^	106.70 ± 0.79^b^	128.57 ± 0.96^a^	125.98 ± 1.71^a^

The results are expressed as the average of three replications and ± represent standard deviations of the mean. Different alphabetical letters on the superscript indicate significant differences between the treatments according to Duncan's multiple range test at p ≤ 0.05. (T1) Tv; (T2) Bs; (T3) Pf; (T4) Tv + Bs; (T5) Tv + Pf; (T6) Bs + Pf; (T7) Tv + Bs + Pf; (T8) Control.

**Table 2D:** Effect of microbes in either individual or consortium treatments on the phenolic content (μg g^−1^ FW) in the leaves of potato plants at 96 h after pathogen inoculation.

Treatments/Phenolics	T1	T2	T3	T4	T5	T6	T7	T8
Shikimic acid	513.30 ± 9.28^a^	500.87 ± 2.35^a^	481.77 ± 15.03^a^	634.36 ± 11.32^a^	596.81 ± 7.54^a^	585.32 ± 12.72^a^	1037.87 ± 9.52^a^	390.10 ± 8.98^b^
Gallic acid	46.54 ± 0.96^g^	53.31 ± 1.78^e^	50.13 ± 1.57^f^	74.14 ± 1.56^b^	61.16 ± 1.75^c^	56.92 ± 2.23^d^	100.93 ± 1.78^a^	39.55 ± 1.14^h^
Kaempferol	0.54 ± 0.03^d^	0.56 ± 0.02^d^	0.54 ± 0.03^d^	1.52 ± 0.04^a^	0.57 ± 0.02^d^	0.55 ± 0.03^d^	0.75 ± 0.04^c^	0.85 ± 0.02^b^
Rutin	74.08± 1.86^f^	64.78 ± 2.18^g^	84.14 ± 1.75^e^	144.32 ± 1.74^a^	103.90 ± 4.27^b^	87.21 ± 1.40^d^	98.44 ± 0.91^c^	103.24 ± 1.74^b^
3,4-Dihydrocinnamic acid	5.10 ± 0.19^e^	5.34 ± 0.11^d^	5.25 ± 0.10^de^	6.26 ± 0.12^a^	5.94 ± 0.10^b^	6.06 ± 0.07^b^	2.07 ± 0.08^f^	5.64 ± 0.10^c^
Tannic acid	76.24 ± 1.71^e^	65.24 ± 2.66^f^	61.30 ± 1.63^g^	123.29 ± 0.75^a^	84.74 ± 1.24^d^	88.46 ± 0.97^c^	105.73 ± 1.12^b^	88.50 ± 1.06^c^

The results are expressed as the average of three replications and ± represent standard deviations of the mean. Different alphabetical letters on the superscript indicate significant differences between the treatments according to Duncan's multiple range test at p ≤ 0.05. (T1) Tv; (T2) Bs; (T3) Pf; (T4) Tv + Bs; (T5) Tv + Pf; (T6) Bs + Pf; (T7) Tv + Bs + Pf; (T8) Control.

### 3.7 Histochemical analysis

#### 3.7.1 Detection of hydrogen peroxide (H_2_O_2_) accumulation in potato leaves

**Fig. 13:**
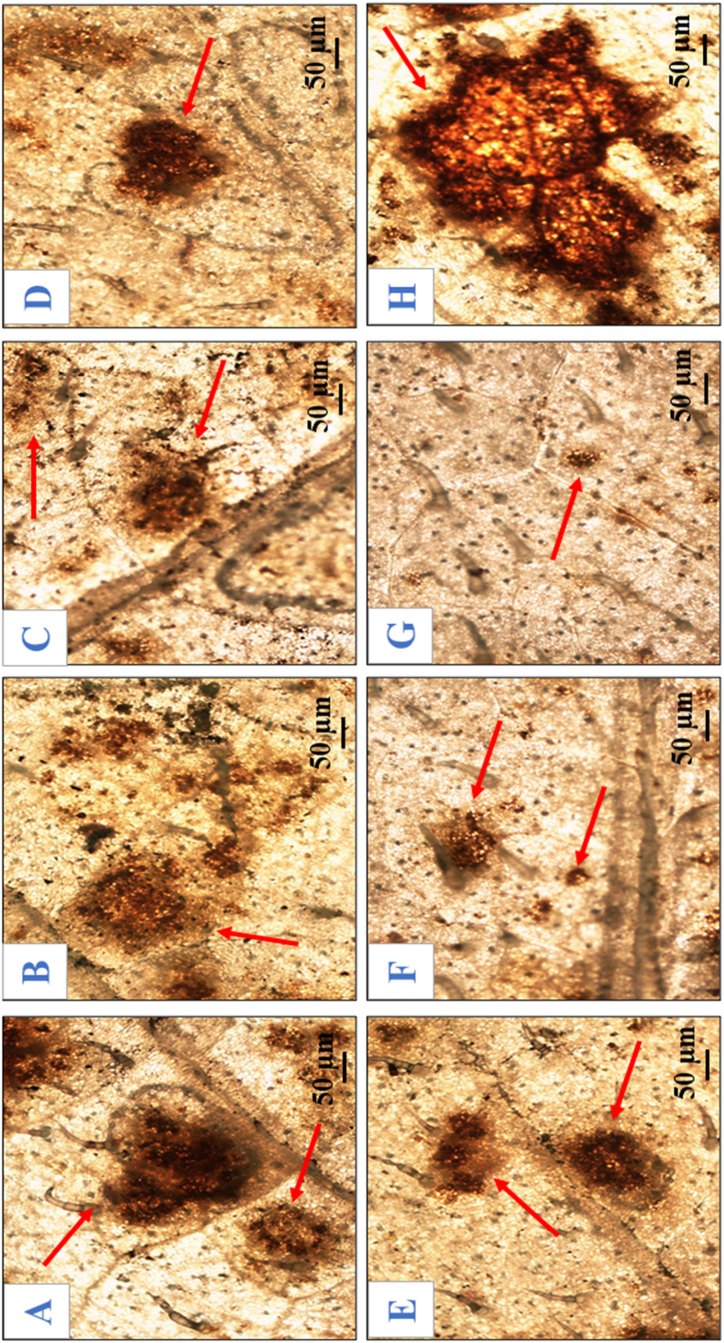
Microscopic detection of the accumulation of hydrogen peroxide (arrow) in potato leaves as visualized by DAB staining at 72 hapi. (A) *Trichoderma viride*; (B) *Bacillus subtilis*; (C) *Pseudomonas fluorescens*; (D) *Trichoderma viride* + *Bacillus subtilis*; (E) *Trichoderma viride* + *Pseudomonas fluorescens*; (F) *Bacillus subtilis* + *Pseudomonas fluorescens*; (G) *Trichoderma viride* + *Bacillus subtilis* + *Pseudomonas fluorescens*; (H) Control.

The microscopic visualization of potent sites generating H_2_O_2_ was observed as dark brown polymerization by using DAB staining (Fig. 13). The main sites of H_2_O_2_ production among all the dead cell lesions developed as dark brown spots on reaction with DAB, and the maximum number of brown spots was found in pathogen-challenged control plants, followed by those plants that were primed with the three-microbe consortium, followed by the double microbe consortium, and individually treated plants inoculated with pathogen. The microscopic observation results revealed that the pathogen-inoculated leaves showed an increased number of brown spots (Fig. 13H), followed by single bioagent-treated plants (Fig. 13A–C). The three species bioagent consortium treated plants challenged with pathogen showed significantly fewer dark brown spots (Fig. 13 G) than the other treatments (Fig. 13 D-F).

### 3.8 Cluster hierarchical analysis revealed the differences between the individual or consortium of biocontrol agents

**Fig. 14:**
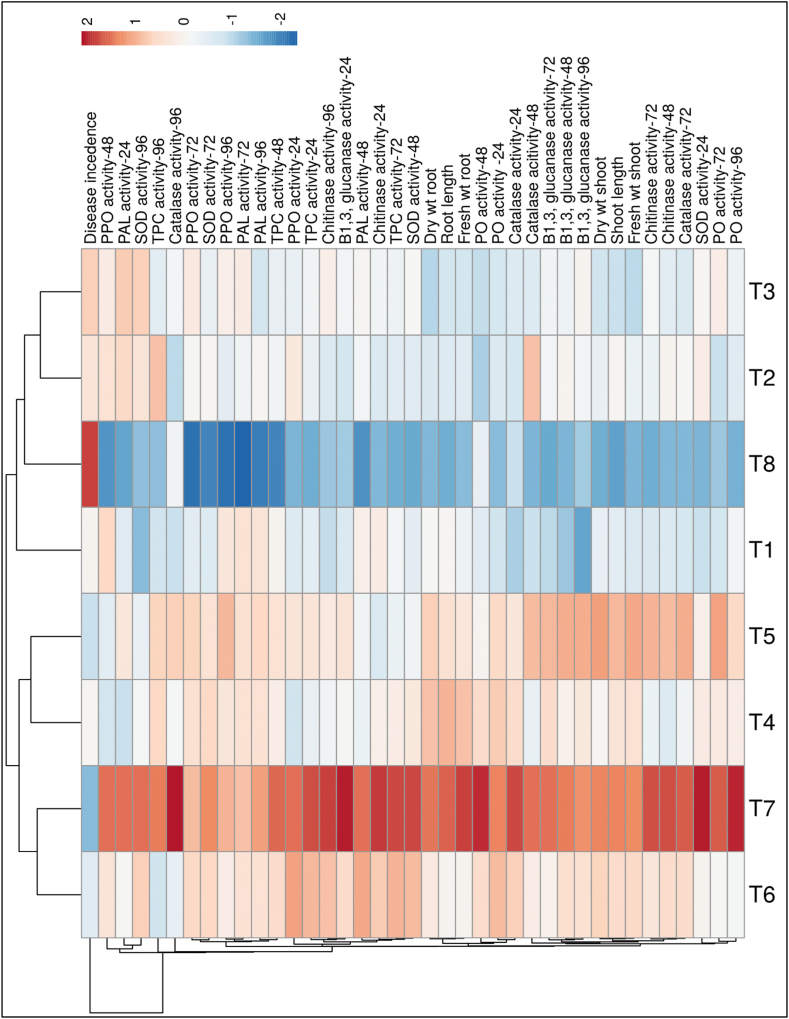
Heatmap and two-way cluster hierarchical analysis. The differences in the response variable between all applied treatments are shown in the heatmap diagram. The rows express the individual response variables, and the columns express treatments such as (T1) *Trichoderma viride*; (T2) *Bacillus subtilis*; (T3) *Pseudomonas fluorescens*; (T4) *Trichoderma viride* + *Bacillus subtilis*; (T5) *Trichoderma viride* + *Pseudomonas fluorescens*; (T6) *Bacillus subtilis* + *Pseudomonas fluorescens*; (T7) *Trichoderma viride* + *Bacillus subtilis* + *Pseudomonas fluorescens*; and (T8) Control. Lower numerical values are blue, whereas higher numerical values are red. The map was generated by using the ClustVis website, https://biit.cs.ut.ee/clustvis/. (For interpretation of the references to colour in this figure legend, the reader is referred to the Web version of this article.)

Additionally, the cluster hierarchical analysis and its related heatmap were created utilizing the individual response variables (Fig. 14). The results revealed that the three-microbe consortium (T7) and a consortium of *B. subtilis* + *P. fluorescens* (T6) were clustered together separately from the pathogen inoculated control plants. The heatmap revealed that higher disease incidence was observed in pathogen-inoculated control plants expressed as red colour and minimum disease incidence was seen in a three-species microbe consortium expressed as blue colour. On the other hand, all growth parameters (i.e., shoot length, root length, shoot fresh and dry weight, and root fresh and dry weight) and plant defense enzymatic activities at different time intervals were observed to be higher in the three-species microbe consortium along with pathogen-treated potato tubers compared to other treatments (Fig. 14).

### 3.9 Principal component analysis (PCA) of treatment variables

**Fig. 15:**
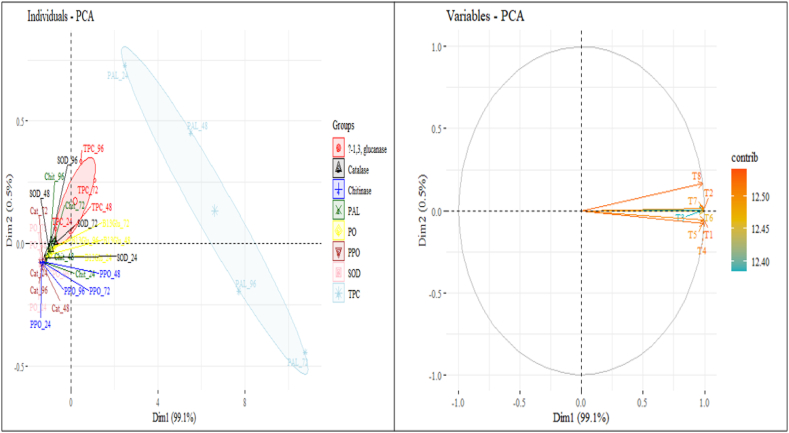
Ordination diagram of principal component analysis (PCA) for variable treatments such as (T1) *Trichoderma viride*; (T2) *Bacillus subtilis*; (T3) *Pseudomonas fluorescens*; (T4) *Trichoderma viride* + *Bacillus subtilis*; (T5) *Trichoderma viride* + *Pseudomonas fluorescens*; (T6) *Bacillus subtilis* + *Pseudomonas fluorescens*; (T7) *Trichoderma viride* + *Bacillus subtilis* + *Pseudomonas fluorescens*; (T8) Control. The circle has a correlation value of 1.0, and the arrow lengths for each treatment are proportionate to the correlation coefficient.

The results are explained by the two principal components (Dimensions 1 and 2) of eight treatment variables (Fig. 15). The eight treatment variables T1, T2, T3, T4, T5, T6, T7, and T8 are positively correlated with one another. On the other hand, as shown in Fig. 14, the arrows of these variables are clustered together, indicating a positive correlation.

## 4 Discussion

In the last few decades, the worldwide human population has increased drastically as well as food demand and pressure on the scientific community to evolve a reliable approach for enhancing sustainable food production without causing harmful effects on ecosystems and human health [75,76]. From this perspective, the use of beneficial microbes evolved as a promising alternative strategy for obtaining high-quality, disease-free food products [77]. To obtain disease-free fruits and vegetables, researchers have investigated the use of various beneficial organisms used as single or as a combination for managing disease-causing agents, such as fungi, bacteria, viruses, and nematodes [[78], [79], [80]]. Recently, microbial consortia have played an important role as an eco-friendly and spectacular method for controlling the disease [[81], [82], [83]]. Microbial consortia are mixtures of compatible but diverse groups of two or more beneficial organisms that have the potential to trigger ISR and provide elevated protection against pathogen invasion along with enhanced plant growth in both field and greenhouse conditions [[84], [85], [86], [87], [88]]. Plants inoculated with microbial consortium aptly enhanced the cellular defense response, such as the upregulation of defense genes and oxidative burst after plants were challenged with the pathogen compared with unchallenged plants [[89], [90], [91], [92]]. In the present study, we included three compatible BCAs as our microbial consortia, namely *T. viride*, *B. subtilis*, and *P. fluorescens*, to assess their impact on plants and to gain deeper knowledge about the mechanism of plant responses to the biotic stress incited by *A. solani* by recording the plant growth promotion activities, disease protection, innate defense responses, and reactive oxygen species management mediated by the microbial consortium. The results obtained from this study aptly encourage an eco-friendly approach to hamper the inimical early blight pathogen *A. solani*.

Growth-promoting microbial consortium is a feasible approach for inducing and promoting the key processes that are helpful for plant growth and development, which is directly linked with the sustainable agricultural practices [[93], [94], [95], [96]]. In our study, the triple microbe consortium used, i.e., *T. viride* + *B. subtilis* + *P. fluorescens*-treated tubers, showed a significant increase in plant growth with respect to the length of shoots and roots, the fresh and dry weights of shoots and roots compared to pathogen-challenged control plants. These findings can be correlated with observations made by Erdemci [97]. As they reported, the seeds of wheat coated with a consortium significantly enhanced plant height and yield. Similar findings were observed by Kumar et al. [98], who suggested that microbial consortiums of *B. subtilis* OTPB 1 and *T. harzianum* OTPB 3 coated tomato seeds showed elevated plant growth and induction of systemic resistance compared to their single-treatment counterparts. Recent studies conducted by Tsalgatidou et al. [99] reported that tomato seed biopriming with a combination of *Bacillus halotolerans* (Cal.l.30 and Cal.l.4) gave better results in plant growth promotion parameters such as shoot and root length and plant biomass compared to untreated plants under pathogen-challenged conditions. In another investigation, the combined treatment of *P. gessardi* EU-LWNA-25, *Bacillus* sp. strain IARI-HHS2-45, and *Erwinia rhapontici* EU-B1SP1 significantly increased the growth promoting activities of *Amaranthus* plants under both greenhouse and field conditions [100]. The results from the current study confirmed that potato plants germinated from tubers inoculated with microbial consortia challenged with pathogen showed significant promotion in overall growth parameters.

In the current study, the potato tubers that were raised with a consortium of three beneficial microbes revealed significant inhibition of percent disease incidence and early blight symptoms compared to control plants inoculated with *A. solani* under greenhouse conditions. Moreover, the potato tubers raised with a triple species of microbe consortium also endowed enhanced protection against *A. solani* infection followed by dual consortia compared to those raised with a single microbe via induction of systemic resistance. Similar observations were recorded by Palmieri et al. [86], who revealed that chickpea plants primed with a consortium comprising four bacterial isolates suppressed *F. oxysporum* f. sp. *ciceris*, which causes *Fusarium* wilt, and enhanced the growth-promoting capacity of chickpea under field conditions. In a recent study, the combination of two biocontrol agents, *T. harzianum* (Th38) and *P. fluorescens* (Pf28), significantly induced resistance against sheath blight disease through seed biopriming in basmati rice [101]. In another study, it was found that seeds bioprimed with bioagents (*T. asperellum* and *T. harzianum*) significantly induce resistance in chilli against anthracnose disease [102]. The current study confirmed that tubers primed with *T. viride* + *B. subtilis* + *P. fluorescens* consortium prevented the ingression of *A. solani* and improved plant growth. The disease tolerance of potato plants against *A. solani* was improved as a result of the induced defense response through the application of a microbial consortium.

Plants that are primed with microbial consortia aptly produce myriad effector biomolecules that enhance the activity of the flux using the phenylpropanoid pathway, where cinnamic acid is formed concomitantly from phenylalanine [103]. Phenylalanine ammonia-lyase (PAL) is an endowed equipped enzyme that plays an important role in the transformation of l-phenylalanine to ammonia and *trans*-cinnamic acid, which is ultimately involved in the biosynthesis of numerous physiologically important secondary metabolites that play noteworthy roles in disease resistance against invading pathogens, such as coumarins, phenols, flavonoids, phenylpropanoids, stilbenes, and lignin with defense functions and drastically linked to the production of signaling molecules such as salicylic acid [[104], [105], [106]]. Plants have the ability to produce various natural compounds like phenolics, which are formed by activation of the phenylpropanoid pathway and are directly associated with the plant's defense against pathogen invasion [107]. A significant increase in PAL activity in the current study was recorded in potato tubers treated with a consortium of three microbial species compared with other microbial consortia and the control treatment, which were challenged with *A. solani*. Similarly, a significant enhancement in total phenolic content (TPC) was also found in plants that were treated with three beneficial microbe consortia compared to the pathogen-challenged control treatment. The current experimental results were supported by the observations of Harman et al. [108], where chilli seedlings primed with a consortium of two species of *Trichoderma* indicated that higher accumulation of phenolic content was observed after pathogen inoculation. Das et al. [93] reported that basmati rice seed biopriming with T. harzianum (Th38) + P. fluorescens (Pf28) significantly suppressed the level of stress markers and subsequently induced defense-related enzyme activities such as PO, PAL, and total phenolics compared to the sheath blight-infected control. A higher accumulation of total phenolic content was observed in bioprimed banana plants as compared to *Fusarium* wilt-infected plants, as reported by Wong et al. [109]. In the current study, increased PAL activity and higher accumulation of phenolic content were found to be directly correlated with enhanced plant defense against pathogen infection.

Pathogenesis-related proteins, also known as PR proteins, are plant species-specific proteins that are toxic to invading catastrophic plant pathogens. PR proteins are a set of novel proteins mainly produced by various plant organs in lower amounts and directly linked with host defense, which hampers the pathogen progression [110,111]. The two important PR proteins, β-1,3-glucanase and chitinase, belonging to the PR 2 and PR 3 families, respectively, play pivotal roles in plant resistance [[112], [113], [114]]. Saravanakumar et al. [115] demonstrated that when rice plants were treated with a consortium of *Pseudomonas* spp., higher accumulations of chitinase were found against the sheath rot pathogen caused by *Sarocladium oryzae*. In our study, the β-1,3-glucanase (PR-2) and chitinase (PR-3) levels were also significantly increased in the tubers of the triple consortia challenged with the early blight pathogen compared with the tubers of the single or dual consortia. These findings were supported by an earlier report by Karthikeyan et al. [116], which reported that integrated application of *P. fluorescens* + *T. viride* in combination with chitin significantly enhanced the β-1,3-glucanase and chitinase activities in coconut palm when compared with control and other treatments. Recently, it has been demonstrated by transcriptomic analysis of the genes encoding the pathogenesis-related proteins showed that genes *PR 2* and *PR 3* was found to be upregulated by *Paenibacillus alvei* K165 and *Blastobotrys* sp. FP12 inoculation in grape berries, which encode the β-1,3-glucanase and chitinase, respectively, which indicates the activation of the defense response against grapevine bunch rot caused by *Botrytis cinerea* [117]. Yadav et al. [102] reported that two pathogenesis-related genes (*PR-2* and *PR-5*) were significantly expressed in chilli bioprimed seeds under *Colletotrichum truncatum*-challenged conditions. The current study confirmed that the PR proteins correlated with oxidative stress could exhibit higher protection against the early blight pathogen *A. solani*.

The first line defense response in the host plant against invading pathogens elevates the production of ROS comprising H_2_O_2_, O_2_^−^, and HO^−^ [118,119]. The excessive release of ROS can cause harmful effects on plant growth and development, damage nucleic acids and major pigments, and cause the oxidation of proteins by disturbing the plant metabolic process [120,121]. To suppress the oxidative stress caused by ROS, plants have the ability to produce antioxidant protective mechanisms for detoxification of the intrinsic effects of ROS [46,122]. The SOD enzyme is the first line of defense against oxidative stress, which causes the dismutation of O_2_^−^ into H_2_O_2_ and O_2_ [123]. Moreover, APX and CAT are two important enzymes that can scavenge H_2_O_2_ [124,125]. In this scenario, it has been experimentally shown that microbial consortia mediate protection against ROS by enhancing the ROS scavenging capacity. In the present investigation, the highest accumulation of H_2_O_2_ was observed in the control treatment challenged with *A. solani*, while the lowest accumulation was found in the microbial consortium. Moreover, to inhibit the harmful effects of ROS molecules, we found that SOD and catalase play a pivotal role against H_2_O_2_, and their activities were higher in potato tubers treated with three species of consortia challenged with pathogen compared with control and other treatments. In the consortium-treated plants, a significant increase in SOD and catalase was found in correlation with a reduction in the accumulation of H_2_O_2._ A recent report indicated that chilli seeds pre-treated with *T. asperellum* + *T. harzianum* induced a significant increase in the antioxidant enzymes such as SOD, CAT, APX, and GPX, and higher accumulation of ROS molecules like H_2_O_2_ and O_2_^−^ was observed in pathogen inoculated leaves [22]. Recently, Farhat et al. [126] reported that rapeseed seeds bioprimed with a consortium of *Rhizobium* spp., *Pseudomonas* spp., and *Bacillus* spp. significantly increased the efficiency of oxidant quenching enzymes such as CAT, SOD, and APX compared to non-treated plants. In another study, Shukla et al. [127] reported that the highest H_2_O_2_ was scavenged by microbial consortium-treated tomato plants (*B. subtilis* and *P. fluorescens*) and the lowest in pathogen-challenged plants compared to the untreated control. They also reported that the accumulation of antioxidant enzymes such as SOD, APX, and CAT was found to be highest in consortium-treated plants and lowest in pathogen-inoculated controls.

The various defense-related enzymes like PO, PPO, and PAL are induced, which provides strong protection for the host plant against biotic and abiotic stress [[128], [129], [130]]. Peroxidases are glycoproteins that are synthesized by the endoplasmic reticulum and play a number of physiological functions in plant resistance via wound healing and plant cell elongation [[131], [132], [133]]. Polyphenol oxidase is a ubiquitous copper-containing enzyme that oxidize phenolics to highly toxic quinones and has a potential role in disease resistance [134,135]. The current experimental results showed that the triple microbe consortium treated tubers induced defense-related enzymes like PO, PPO, and PAL compared to the control treatment and other treatments. In a recent study, it was observed that leaves of brinjal plants pre-treated with a consortium of *Trichoderma* spp. challenged with *S. sclerotiorum* showed significantly higher defense related enzymes like PO, PPO, and PAL activities [136]. Various other studies mentioned the synergistic use of the triple consortium of *Trichoderma*, *Pseudomonas*, and *Rhizobium* [137] and the potential dual consortium of *T. viride* + *T. erinaceum* [138], which significantly enhanced the defense enzymes like PAL, PO, and PPO provided protection against inimical plant pathogens. Recently, Abbasi et al. [139] showed an increase in defense enzymes like PO, PPO, and PAL in pepper seeds bioprimed with *Streptomyces* strains (SS14 and IT20) compared to untreated control. In the present study, beneficial microbes, either individually or in combination, not only stimulated the plant growth but also concomitantly enhanced the phenylpropanoid and shikimic acid pathways, which provides elevated potato plant protection against *A. solani* infection as determined by HPLC phenolic analysis. When the potato plants were inoculated with microbes and inoculated with *A. solani*, we targeted six phenolics, namely, shikimic acid, gallic acid, kaempferol, rutin, 3,4-dihydrocinnamic acid, and tannic acid. Shikimic acid plays a pivotal role in the biosynthesis of myriad phenolic compounds such as ferulic acid, cinnamic acid, and phenylalanine, which finally induce lignification in host plants [140,141]. Singh et al. [142] reported that t-cholorogenic acid (CHA), ferulic acid, and protocatechuic acid are potential phenolics that have promising antifungal activities and drastically enhance the defense system of the plant in response to invading pathogens.

In our study, we observed that three potential phenolic compounds like shikimic acid, rutin, and tannic acid were much more abundant than the other three phenolics, which play a potent role in plant defense (Table 2A, Table 2B, Table 2C, Table 2DD). Three-microbe species consortia, followed by dual species and challenge with *A. solani*, expressed the maximum shikimic acid amount when compared with single species of beneficial microbes and pathogen-inoculated control. Several times the increase in shikimic acid, which helps in the synthesis of different plant phenolics, in the microbe consortium indicated the role of the shikimic acid pathway in combating pathogen ingress [[143], [144], [145]]. We assume that the elevated accumulation of shikimic acid in the BCAs primed potato plants, particularly in triple or dual consortia after *A. solani* inoculation, demonstrated the activation of ISR, where the shikimic acid pathway plays a pivotal role in the synthesis of antipathogen compounds. The same results were observed by Bisen et al. [146], who demonstrated that brinjal plants treated with *Trichoderma* BHU51+BHU105 consortium challenge with *Sclerotium rolfsii* significantly induced shikimic acid accumulation. Moreover, rutin and tannic acid were significantly induced in consortium-treated plants after being infected with *A. solani* in the present study, demonstrating that they may provide enhanced protection against early blight. Rutin plays a pivotal role in early plant defenses which are directly correlated with the suppression of the progress of the pathogen in the host plant. The higher augmentation of rutin in the potato plants in our current study also correlated with a previous study conducted by López‐Gresa et al. [147]. Recently, Singh et al. [148] reported that tomato seeds treated with a consortium of *T. harzianum* + *T. asperellum* significantly increased the concentration of shikimic acid among eight phenolic compounds under *Sclerotinia sclerotiorum*-challenged conditions.

Flavonols, which belong to the flavonoid subclass, such as kaempferol, quercetin, and isorhamnetin, are available in both free and glycosylated forms in different plant species, which hampers pathogen infection because they have antimicrobial properties [[149], [150], [151]]. Kaempferol is an important flavonoid that works as an antioxidant and protects stressed plant cells by scavenging H_2_O_2_ [152,153]. In the present study, the kaempferol content was significantly higher in triple-microbe species consortia treated plants challenged with pathogen, demonstrates that at the site of *A. solani* infection, the cells were more stressed, and the applied treatment of BCAs had the capacity to alleviate the stress. Our current results have also been supported by the observations of Jain et al. [154], where pea plants primed with consortia of bioagents inoculated with *S. sclerotiorum* significantly induced kaempferol content, which alleviates the biotic stress caused by plant pathogens. From the current study, it is clear that higher accumulation of phenolic compounds and induction of defense-related and antioxidant enzymes were observed in consortium-treated plants, which are potentially equipped to suppress early blight incidence via induction of systemic resistance.

## 5 Conclusion

In this context, the interesting outcomes of the present investigation suggested that potato seeds treated with a consortium of three microbes, *T. viride*, *B. subtilis*, and *P. fluorescens* induced numerous defense mechanisms against *A. solani* infection. Moreover, the lowest disease incidence was found in the triple microbe consortium, followed by the dual microbe consortium. The biocontrol microbe consortium not only induces defense systems in potato but also enhances plant growth promotion activities. Additionally, potato tubers primed with the consortium and inoculated with pathogen exhibited a significant increase in total phenolic content, defense-related enzymes, PR proteins, and induced antioxidant enzymes compared to the control plants challenged with *A. solani*. The accumulation of ROS like H_2_O_2_ was highest at 72 hapi in pathogen inoculated controls and lowest in consortia. In conclusion, this study demonstrates that microbial consortia enhances plant resistance against *A. solani* infection by interacting in a synergistic manner thereby, providing better opportunities for solving agricultural challenges and sustainable management of early blight of potato.

## Ethics approval and consent to participate

Not Applicable.

## Consent for publication

All authors have read and agreed to the published version of the manuscript.

## Data availability statement

The datasets generated during and/or analyzed during the current study available from the author S.K. on request.

## Funding

C.K. gratefully acknowledges the financial support from the project of the Ministry of Science and Higher Education of the Russian Federation on the Young Scientist Laboratory within the framework of the Interregional scientific and educational center of the South of Russia (no. LabNOTs-21-01AB, FENW-2021-0014) and the Strategic Academic Leadership Program of the Southern Federal University (“Priority 2030”).

## CRediT authorship contribution statement

**Sumit Kumar:** Data curation, Formal analysis, Investigation, Methodology, Writing – original draft. **Ram Chandra:** Conceptualization, Resources, Supervision, Writing – review & editing. **Lopamudra Behera:** Data curation, Formal analysis, Investigation, Methodology, Writing – original draft. **Ichini Sudhir:** Formal analysis, Investigation, Methodology, Writing – original draft. **Mukesh Meena:** Methodology, Software, Validation, Visualization, Writing – review & editing. **Shailendra Singh:** Data curation, Methodology, Validation, Writing – review & editing. **Chetan Keswani:** Conceptualization, Funding acquisition, Methodology, Project administration, Resources, Writing – review & editing.

## Declaration of competing interest

The authors declare the following financial interests/personal relationships which may be considered as potential competing interests:Chetan Keswani reports financial support was provided by 10.13039/501100012190Ministry of Science and Higher Education of the Russian Federation.
